# Nanotechnology as a Platform for the Development of Injectable Parenteral Formulations: A Comprehensive Review of the Know-Hows and State of the Art

**DOI:** 10.3390/pharmaceutics12060510

**Published:** 2020-06-03

**Authors:** Maryam A. Shetab Boushehri, Dirk Dietrich, Alf Lamprecht

**Affiliations:** 1 Department of Pharmaceutics, Faculty of Pharmacy, University of Bonn, 53121 Bonn, Germany; alf.lamprecht@uni-bonn.de; 2 Department of Neurosurgery, University Clinic of Bonn, 53105 Bonn, Germany; dirk.dietrich@ukbonn.de; 3 PEPITE EA4267, Institute of Pharmacy, University Bourgogne Franche-Comté, 25000 Besançon, France

**Keywords:** nanotechnology, injectable parenteral formulations, solubility enhancement, controlled release, targeting, adjuvancy, immune activation

## Abstract

Within recent decades, the development of nanotechnology has made a significant contribution to the progress of various fields of study, including the domains of medical and pharmaceutical sciences. A substantially transformed arena within the context of the latter is the development and production of various injectable parenteral formulations. Indeed, recent decades have witnessed a rapid growth of the marketed and pipeline nanotechnology-based injectable products, which is a testimony to the remarkability of the aforementioned contribution. Adjunct to the ability of nanomaterials to deliver the incorporated payloads to many different targets of interest, nanotechnology has substantially assisted to the development of many further facets of the art. Such contributions include the enhancement of the drug solubility, development of long-acting locally and systemically injectable formulations, tuning the onset of the drug’s release through the endowment of sensitivity to various internal or external stimuli, as well as adjuvancy and immune activation, which is a desirable component for injectable vaccines and immunotherapeutic formulations. The current work seeks to provide a comprehensive review of all the abovementioned contributions, along with the most recent advances made within each domain. Furthermore, recent developments within the domains of passive and active targeting will be briefly debated.

## 1. Introduction

Though the word “parenteral” terminologically refers to the routes of administration that avoid the alimentary canal, parenteral delivery in today’s health care system mostly involves the injection of the drug through intradermal, subcutaneous, intramuscular, intravenous and intra-arterial pathways. Adjunct to the injectable formulations, parenteral dosage forms also include biodegradable implants, transdermal patches, and ocular delivery systems [1]. The focus of the current review, however, will be mainly the injectable systems commonly used for drug delivery purposes.

Notwithstanding the invasiveness, injection remains an indispensable route of delivery for a wide range of active pharmaceutical ingredients (APIs). In addition to advantages such as the rapid onset of action, possibility to administer a mixture of APIs, and convenience for hospitalized patients with special conditions (e.g., unconscious or orally restricted patients), parenteral administration is associated with a wide range of benefits, such as avoiding the hostile gastrointestinal environment, possibility to deliver macromolecular APIs with low gastrointestinal absorption (e.g., proteins and peptides), circumventing the hepatic first pass metabolism, and potential to achieve an extended duration of the therapeutic effect [2,3].

Conventionally, injectable parenteral dosage forms can be formulated as solutions, suspensions or emulsions. The advent and development of nanotechnology, however, has introduced new opportunities to improve the efficiency and elaborate the potentials of these conventional dosage forms [1]. A variety of benefits justify the application of nanoparticulate systems for injection-based parenteral drug delivery. These include enhancing the solubility of poorly water-soluble actives, thus improving their bioavailability, developing prolonged release parenteral depots, facilitating targeted delivery to specific organs, tissues, cells, or even organelles, and protecting the incorporated cargo from the harsh extra- and intracorporeal conditions [4,5,6,7]. The present review seeks to elaborate on the application of nanostructures for injection-based parenteral drug delivery and the various platforms created within this context. A list of marketed injectable nanomedicine is tabulated in Table 1, while the injectable nanoparticle-based therapeutic formulations going through various stages of clinical trials are presented in Table 2. A significant number of the nanosystems within each category highlights the rapidly growing role of nanotechnology within the domain of injection-based drug delivery.

## 2. Solubility Enhancement 

One of the early grounds upon which the first injectable nanosystems were developed was to enhance the solubility of the drugs with limited hydrophilicity. Low water solubility is a major challenge restricting the clinical translation of a remarkable number of actives. According to the estimates, as high as 40% of the marketed drugs and 90% of the pipeline products suffer from low aqueous solubility issues [11]. The Biopharmaceutics Classification System (BCS) defines poorly water-soluble actives as drugs whose highest dose strength is insoluble in a maximum volume of 250 mL aqueous medium over the pH range of 1 to 7.5 [12]. These include both the lipophilic BCS class II and the lipophobic BCS class IV molecules [13]. A wide range of approaches has been proposed to enhance the solubility of such actives. Despite offering special merits, each of these commonly used strategies is associated with certain limitations. These include temporariness of the effect when the lattice structure of the active is modified, alteration of the pharmacological activity in case the chemical structure of the API is manipulated, and toxicity issues should high concentrations of solubilizing excipients be used [14]. Moreover, the intended route of administration can impose further challenges. Examples are inappropriateness of some formulation vehicles for injection purposes, reactions at the injection site in case the pH modification or salt formation strategies are used, precipitation of the drug following the intravenous injection of the co-solvent systems, and dissociation of the drug-cyclodextrin complexes under the effect of dilution in plasma [15,16]. Hence, as an alternative to such approaches, nanotechnological strategies have been gaining ever-growing interest. These strategies have ample to offer for the development of intravenously injectable formulations of low water-soluble drugs. Not only can such strategies help to overcome the low water solubility issues, they can also serve the purpose of targeted delivery or controlled release of the incorporated cargo.

**Figure 1:**
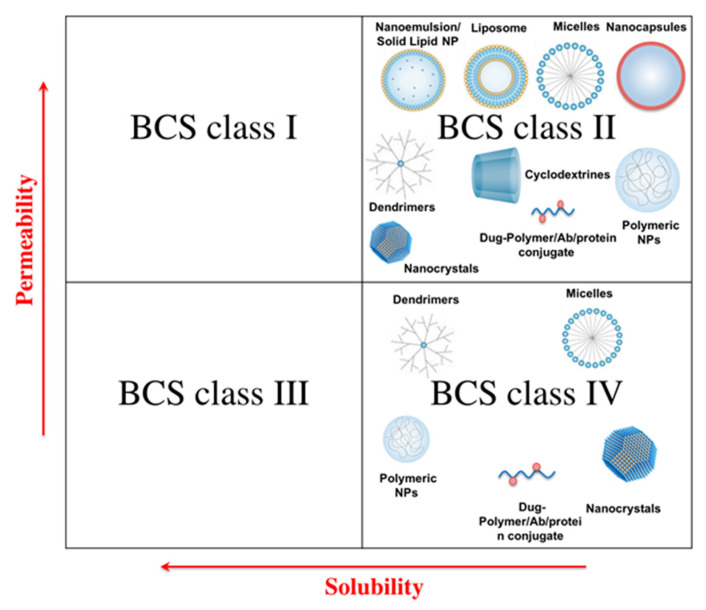
Overview of different types of nanocarriers applicable for solubility enhancement of various classes of low water-soluble drugs.

Depending on the properties of the payload and the additional delivery considerations and requirements, various types of nanocarriers have been exploited to address low water solubility issues of different APIs. An overview of these has been presented in Figure 1. For instance, BCS class II APIs are often incorporated within lipophilic nanostructures such as nanoemulsions, liposomes, solid-lipid nanoparticles (SLNs), and sometimes micelles. The important issue within the context of formulating such nanoparticles, particularly when the system is to be intravenously injected, is the optimization of the drug release to avoid premature precipitation or the interminable entrapment of the drug within the carrier. The former occurs in case of the low affinity between the drug and the carrier, or the suboptimal formulation of the particulate system. For instance, avoiding the premature leakage and precipitation of the cargo in case of the nanoemulsion formulations necessitates a log P greater than 9 to ensure adequate lipophilicity [17], which is of course quite rare. Similarly, unless properly formulated, the cargo can be prone to premature expulsion and precipitation due to phenomena such as the increase of liposomal bilayer permeability following the drug incorporation [18], polymorphic transformation in case of the poorly formulated SLNs [12], and dissociation of the classical surfactant-based micelles upon dilution in biological fluids [19]. Conversely, too strong an affinity between the drug and the hydrophobic carrier might account for long-term intraparticulate retention, leading to accumulation in certain tissues, organs or cells [4,17]. This drawback can be of course exploited to achieve a passive targeted delivery of the incorporated active to the site of action, e.g., inflamed tissues or tumor site.

Polymer-based systems create a further platform to ameliorate the solubility of low water-soluble APIs. The most common polymeric systems used within this frame include the polymeric nanospheres and nanocapsules, drug-polymer conjugates, polymeric micelles and dendrimers. While hydrophobic polymers can be used to entrap or encapsulate lipophilic BCS class II compounds, direct conjugation of the hydrophobic BCS class IV APIs to the polymeric chains using cleavable bonds provides further opportunities to enhance the solubility thereof. Linear polymers conjugated to chemotherapeutic drugs have been perhaps the most fascinating candidates to improve the water solubility of these agents, while enabling their targeted delivery to the tumor site. The most common of the polymers used for this purpose include poly(ethylene glycol) (PEG), N-(2-hydroxypropyl)methacrylamide (HPMA) copolymers, dextran, poly L-glutamic acid (PGA), and carboxymethyldextran, [20,21]. Several polymer-drug conjugates are currently under clinical trials and will hopefully find their way to the pharmaceutical market [22] (see Table 2). Polymeric micelles are formed from amphiphilic polymers, and due to their relatively low critical micelle concentrations (CMC), are associated with significantly higher stability than their conventional surfactant-based counterparts [11]. Low water-soluble cargos can be either directly conjugated to the polymer chains prior to micelle formation, or else accommodated within their hydrophobic core following preparation. Despite benefits associated with conjugation approach, e.g., higher loading capacity and better control of the drug release rate, encapsulation still remains favorable due to the simplicity and preservation of the drug composition and physicochemical properties, which will in turn facilitate the characterization and regulatory approval processes [23]. Another type of polymer-based formulations are dendrimers, i.e., tree-like polymeric nanostructures with a single hydrophobic core and numerous hydrophilic branches, which possess “container” properties in solution and are apt to exhibit micelle-like behavior [24,25]. Being associated with advantages, such as high stability (e.g., compared to polymeric micelles) and controllability of their architecture, dendrimers are particularly convenient to enhance the solubility of different APIs [26].

Inclusion complexion of hydrophobic drugs with cyclodextrins is another popular approach to improve their water solubility. From a structural perspective, cyclodextrins are cyclic glucosaccharides comprised of glucopyranose units connected via 1,4-linkage, and are classified as α, β and γ based on the number thereof [27]. While having a hydrophilic exterior, the spatial conformation of cyclodextrins builds up a hydrophobic central cavity, wherein certain “guest” actives of hydrophobic nature can be accommodated [28]. Biodegradability and biocompatibility of the cyclodextrins along with their safety and low toxicity renders them ideal excipients for parenteral delivery [29]. Nevertheless, the main drawbacks of cyclodextrin-based formulations are the instability of the complex during administration and problems arising from their contact with biological media, such as drug explosion, particle size alteration, gelation, etc. [30]. Several approaches have been used to overcome the abovementioned limitations. One strategy is to incorporate cyclodextrin-based complexes within various nanostructures. This would allow combining the advantages of both cyclodextrin and nanoparticle-based technologies, while resolving the issues associated with each. The combination of the two technologies can for instance facilitate the encapsulation of inherently hydrophobic drugs within the aqueous reservoir of liposomes, improving thereby both the loading efficiency of the active and the stability of the drug-cyclodextrin complex [31]. Cyclodextrin-based complexes have been likewise used for the development of various types of nanostructures, such as nanosponges [32,33], host–guest supramolecular vesicles [34,35], niosomes [36], micelles [28], magnetic nanoparticles [37], nano-assembled delivery systems [38], etc. To enhance the solubility of the hydrophobic agents using cyclodextrin-based nanostructures is a novel and promising approach, which will hopefully open door to new possibilities for the development of multipotent carrier systems for injection-based drug delivery.

Yet another interesting approach to enhance the solubility of low water-soluble APIs is their conjugation with different proteins. Albumin is one of the most popular proteins used for this purpose, to which the low water-soluble drugs can be either chemically conjugated or else adsorbed [39]. Additionally, the specific physicochemical properties and extreme robustness of albumin molecules enable their exploitation for the preparation of a variety of nanoparticulate systems through approaches such as coacervation, emulsification, nab technology, self-assembly, and spray drying [40]. The most abundant protein within the human plasma, albumin offers a wide range of advantages for drug delivery and has been of particular interest for the targeting of low water-soluble anticancer drugs to the tumor tissue [41]. The most well-known example is of course Abraxane©, the albumin-bound paclitaxel formulation, which has already found its way to the pharmaceutical market (Table 1) [12].

Apart from the use of nanosized carriers for solubility enhancement purposes, the field of parenteral delivery has further benefited from the advent of the nanocrystal technology. Crystalline nanosized drug particles, nanocrystals address the low solubility issues mainly through the enlargement of the surface area to volume ratio [42], which leads to an increased saturation solubility and dissolution rate of the API [43]. To facilitate parenteral administration, drug nanocrystals can be formulated as nanosuspension, wherein the drug nanocrystals are dispersed in an aqueous milieu often containing stabilizers, or else nanoemulsions, in which the drug molecules are incorporated into the interfacial layer of emulsion-based formulations [16]. Advantages of nanocrystal technology for parenteral delivery, in particular intravenous injection, include a lack of organic solvents or harsh excipients, minimization of the macrophage uptake, high drug loading due to carrier-independent nature of the system, and compatibility with aseptic preparation and sterilization techniques (e.g., sterile filtration, heat treatment and gamma radiation) [16,44]. The production of nanocrystal formulations is achieved through either “bottom-up” or “top-down” approaches, although sometimes combination of both technologies is applied. The “bottom-up” approaches involve the precipitation of the drug nanocrystals from a solution, achieved either through the addition of a non-solvent, or else via alternative strategies such as supercritical fluids, ultrasonic waves or controlled solvent evaporation. “Top-down” technologies seek to reduce the particle size of the active through processes such as wet milling or high pressure homogenization [45]. A detailed review of the nanocrystal production technologies has been presented elsewhere [46]. Though many marketed nanocrystals are intended for oral delivery of the low water-soluble actives, literature holds many examples wherein nanocrystal technology has been exploited for solubility enhancement of APIs for injection purposes. Examples of such APIs include puerarin [47], iron oxide [48], itraconazole [49], paclitaxel [50,51,52], Bexarotene [53], 1,3-dicyclohexylurea [54], nimodipine [55], etc. Furthermore, several nanocrystal formulations have already found their way to the market for parenteral applications (see Table 1) [56]. Hence, nanocrystal technology is growing as one of the most promising nanotechnology-based approaches for solubility enhancement purposes.

## 3. Modification of the Drug Release

Nanocarriers can be formulated to tune the rate or the onset of the cargo’s release. Since these features require different formulation considerations, they will be separately discussed in the following.

### 3.1. Nanoparticles for the Development of Long-Acting Injectables

From the patient’s perspective, invasiveness renders parenteral injectables less favorable than their alternative non-invasively administered counterparts. Nonetheless, injection-based delivery is an indispensable route of administration for a variety of APIs. At the forefront of this approach are macromolecular drugs, such as monoclonal antibodies and recombinant proteins, many of which possess remarkably short elimination half-lives ranging from minutes to hours [57,58]. Hence, reduction of the dosing frequency through the development of long-acting parenteral depots can be beneficial not only to enhance the patient compliance, but also to ameliorate the therapeutic efficiency, reduce the associated side effects and improve the economical aspects of the therapy and manufacturing [59,60]. Conventionally, the development of long-acting formulations is achieved by limiting the absorbable drug concentration either through the deceleration of the cargo’s dissolution or its association with adsorbent molecules. Establishing encapsulation-based barriers to drug release and development of pro-drugs are further strategies to develop injectable depot systems [6,61]. The advent and development of nanotechnology has indeed made a significant contribution to such conventional strategies. Within this context, a wise formulation of the nanoparticles can both enable a sustained release of the drug from the dosage form (control of the drug absorption), and increase the cargo’s bioavailability and elimination half-life (control of the drug excretion). Nanoparticulate prolonged release injectables are either administered through the classic subcutaneous and intramuscular pathways, or else are directly injected into the systemic circulation. Given the specific considerations required for the design of the nanoparticle formulations designated for each of the abovementioned administration routes, a thorough discussion of these will be separately presented in the following sections.

#### 3.1.1. Locally Injectable Long-Acting Nanoparticle-Based Formulations

**Figure 2:**
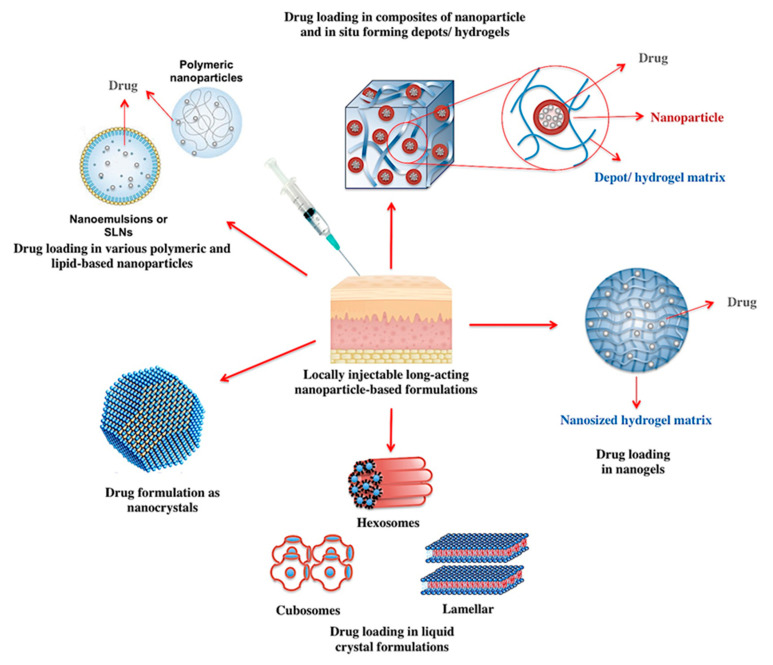
Different platforms where nanotechnology has contributed to the development of locally injectable long-acting formulations.

Subcutaneous or intramuscular injection of the drug solution, emulsion or suspension is a classic approach to achieve a retarded systemic penetration. The systemic release of the drug molecules in this case involves their dissolution within the tissue fluids, followed by their traverse through the interstitium to reach the blood or lymphatic capillaries [62]. This will create a retarded systemic release per se, particularly in case of subcutaneous injection, where lower vascularization levels are in hand [63]. Both the rate of the described process and the fate of the drug molecules are functions of the API’s molecular weight and size. In general, small molecules of less than 1 kD can successfully complete the journey by ending up in the blood capillaries [64]. On the other hand, macromolecules smaller than 16 kD as well as nanocarriers smaller than 100 nm are cleared from the tissue by the lymphatic vessels, while larger particles often remain at the site of injection [62]. The knowledge of the abovementioned alone justifies the encapsulation of the drug molecules within the large nanoparticulate or else microparticulate carriers for the development of extended release locally injectable systems [63]. Due to their smaller surface area to volume ratio, microparticles are more logical candidates for this purpose, and have already found their way into the pharmaceutical market as long-acting formulations [60]. Nonetheless, nanocarriers are associated with more favorable characteristics for injection-based delivery, such as better syringeability and injectability profiles, and ability to deliver a considerable amount of active without drastically impacting the viscosity of the system [58,65]. Consequently, nanoparticle-based formulations are fascinating candidates for the development of long-acting locally injectable systems (Figure 2).

Drug solutions in oily vehicles provide well-established opportunities for localized prolonged delivery of the lipophilic actives [66]. The sustained release of the drug, however, does not seem to be an inherent function of the oily injection base, but the result of the lipophilic interactions between the drug molecules and the lipid-based vehicle [63]. Oil-based long-acting depots are associated with a number of shortcomings, such as potentially long-lasting pain and irritation at the site of injection, which reduce patient compliance [67]. Moreover, the injection locus is often limited to large muscles such as deltoid, triceps, gluteus maximus and rectus femoris, which renders the self-administration of the chronically dosed medications challenging [68]. To overcome such limitations, the encapsulation of lipophilic drugs within the hydrophobic lipid- or polymeric-based nanoparticulate matrices has been alternatively introduced. This strategy offers the unique advantage of injecting lipophilic APIs within the context of an aqueous formulation, while maintaining the lipophilic interactions between the drug molecules and the carrier [63]. In fact, long-acting nanoparticulate systems such as polymeric nanospheres [69,70], SLNs [71], nanoemulsions [72], and liposomes [73] have already been developed for subcutaneous or intramuscular injection and tested in animal models.

Adjunct to ensuring a prolonged release of the drug from the injection site, localized injection has been also exploited to obtain an extended systemic release of the “drug-loaded nanocarriers” as an entire entity [74]. It is worth noting that the size of the nanocarrier in this case should be rigidly controlled to ensure the penetration of the particles in the systemic circulation through either the blood or lymphatic capillaries.

An additional advantage of nanosystems within this frame is their ability to simultaneously prolong the release of a multitude of APIs. While conventional polymeric and lipid-based nanostructures allow for the encapsulation of a cocktail of drugs with similar lipophilic properties [75], incorporation of the APIs with different polarities can be achieved using core-shell nanoparticles [76]. In core-shell nanosystems, the inner core and outer layers can be selected from organic or inorganic materials with different properties [77]. A well-established example is lipid–polymer hybrid core-shell nanoparticles, in which a polymeric core is surrounded by a lipid shell, or vice versa, which allows for the accommodation of molecules with different polarities [78,79]. In this case, the rate of drug release from the particles is determined by the amount of lipid coverage or the number of surrounding polymeric layers [80,81].

Actives with low water solubility can be also locally administered as nanocrystal formulations. Albeit nanocrystals lead to a rapid dissolution of the drugs when subjected to sink conditions, the presence of limited amount of fluids under the subcutaneous and intramuscular conditions can account for a sustained release behavior [45]. For instance, intramuscular injection of memantine-pamoic acid salt and andrographolide nanocrystals resulted in a 3–4 week-long sustained release profile in rats [82,83]. Similarly, a long-acting intramuscularly injectable formulation of rilpivirine nanocrystals could maintain a prolonged release of the drug for three months in dogs and three weeks in mice [84]. Nevertheless, special considerations are required to ensure the stability of such systems following localized injection, which can be negatively impacted by the release of the stabilizers along with the occurrence Ostwald ripening [85].

Another platform in prolonged drug delivery to which nanotechnology has significantly contributed is the field of in situ forming depot formulations. In principle, these systems are liquids with appropriate syringeability, which form (semi)-solid networks upon localized injection [3]. Based on the type and the solidification trigger, these systems can be classified in three major categories of in situ cross-linked systems (photo-initiated polymerized systems, physically cross-linked systems and chemically cross-linked systems), in situ phase separation systems (pH-induced gelling system, thermally induced gelling systems, thermoplastic pastes and systems based on phase separation by solvent exchange), and in situ solidifying organogels [86,87]. Composites of nanoparticles and in situ forming depot formulations can help overcome the limitations associated with both systems. While the solidification of the network can reduce the burst release of the drug from the embedded nanocarriers, nanoparticles can improve the mechanical properties of the depot systems and increase their structural diversity [88]. Additionally, drug-loaded nanoparticles can be designed to undergo cross-linkage in contact with physiological fluids, creating an in situ depot system upon localized injection [89,90].

Among various in situ forming depot systems, hydrogels have been perhaps most widely investigated. As water-insoluble polymers forming three-dimensional cross-linked networks upon injection, hydrogels are able to absorb considerable quantities of aqueous biological fluids [91]. The degree of cross-linkage determines the porosity of the hydrogel matrix, which critically influences the rate of drug release from the system [60]. Depending on the pore size of the polymeric network, hydrogels are classified as macroporous, microporous, and nanoporous systems. While hydrogels can be designed to release their entrapped cargo in a diffusion-, swelling- or chemically-controlled manner, drug release from nanoporous hydrogel networks is mainly governed by diffusion [92,93]. Hydrogels have been of particular interest as depot reservoirs for the sustained delivery of macromolecular drugs, such as proteins, peptides and nucleic acids [94].

Nanotechnology has contributed to the development of prolonged release hydrogel systems in two different ways. The first approach involves the development of hydrogel-nanoparticle composites, which help combine the advantages of both systems. Within this frame, the hydrogel matrix serves to protect the integrity of the nanoparticulate systems while preventing the burst release and further limiting the systemic penetration of the incorporated therapeutic cargo [95]. Nanoparticles on the other hand enable a uniform distribution of hydrophobic drugs within the hydrogel network, reinforce its mechanical stability and endow the system stimuli-responsiveness and multifunctionality [96,97]. Incorporation of the nanoparticles within the hydrogel matrix can be achieved through a variety of strategies. These include the induction of post-injection sol-gel transition within the nanosuspension, physical embedment of the nanocarriers within an already formed hydrogel matrix, reactive hydrogel-mediated formation of the nanoparticles following injection, cross-linkage of the nanoparticles to form hydrogel networks, and hydrogel formation based on polymer-nanoparticle interaction [88,98,99]. Nanoparticle-hydrogel composites have been successfully exploited for the controlled release of the therapeutic cargos such as insulin [100,101], calcein [102] and bone morphogenetic protein 2 (BMP-2) [103]. Furthermore, the sustained release of the entrapped nanocarriers from the hydrogel system has been also achieved [104,105].

A second approach reconciling the nano- and hydrogel technologies is the development of nano-scaled hydrogel, in other words nanogel, systems. Combining the advantages of both parent technologies, nanogels offer numerous advantages such as hydrophilicity, biocompatibility, versatility, flexibility, high loading capacity, controlled release properties, and high water absorptive properties of the hydrogel systems and the nanoparticle-related targeting and multifunctionalization potentials [106]. Nanogels can ensure a prolonged release of the incorporated drug following localized or systemic injections, and can be formulated to trigger the release of the cargo in response to internal or external stimuli [107]. The latter ability will be further discussed under the corresponding section.

Four different classes of polymers have been hitherto exploited for the preparation of nanogels. These include polyacrylates, poloxamer or polyethylene glycol, polypeptides and polysaccharides [108]. One of the most widely investigated nanogel systems for sustained drug delivery are self-assembled hydrophobized polysaccharides, such as cholesterol-bearing pullulan (CHP) [109]. Subcutaneous injection of these nanogels have been used for prolonged delivery of cytokines [110] and protein antigens [111,112]. As an alternative strategy, CHP modified nanogels embedded in hydrogel formulations have been developed, which exhibited a sustained release of the incorporated protein-complexed nanogel [113]. In addition to the CHP nanogels, the ability of N-isopropylacrylamide-based nanogels for prolonged drug delivery following localized injection has been established. These systems have been shown to maintain an extended duration of local anesthesia in rats when loaded with bupivacaine [114]. Further, when injected in the vicinity of the tumor, 5-fluorouracyl-loaded N-isopropylacrylamide-based nanogels were shown to significantly prolong the mean residence time of the drug at the site of injection [115]. Given the diverse potentials of nanogels for parenteral drug delivery, these systems are expected to be subjects of more extensive research in this arena in the future.

Another platform for the development of injectable sustained release systems involves the use of amphiphilic polar lipid molecules that can self-assemble in contact with excess water to form viscose liquid crystalline formulations [116]. Depending on the nature of the lipid or lipid mixture used for their preparation, the water content of the system, the presence of additives, and the solution conditions, such as pH, ionic pressure, and temperature, these systems can assume a number of well-defined geometrical arrangements [117]. These include rod-like lyotropic, lamellar, cubic, and hexagonal liquid crystalline systems [118]. Regardless of the geometrical arrangement, the inner structure of these systems include aqueous and lipidic regions with the potential to provide a slow release matrix for the accommodated hydrophilic or hydrophobic drug molecules [119]. The release of hydrophilic drugs is governed by their diffusion through the water channels and is affected by the composition of the system and the temperature, whereas the diffusion of lipophilic drugs is further dictated by their partition coefficient [120]. While liquid crystalline formulations can be formulated as in situ forming organogel depot systems [121], their dispersion in excess water can form submicron colloidal suspensions [116]. On top of different liquid crystalline nanostructures, cubic and hexagonal nanoparticles, namely cubosomes and hexsosomes have been the paramount subjects research for the development of sustained release parenteral depots [116,122]. Adjunct to their controlled release properties, cubosomes and hexosomes offer ample of further advantages such as improvement of the cargo’s bioavailability, stability and penetrability, and possibility to control the release onset in response to internal or external stimuli [123]. Liquid crystalline nanostructures have been successfully exploited for the sustained localized delivery of various APIs, including leuprolide (luteinizing hormone-releasing hormone analogue) [124], imiquimod and monophosphoryl lipid A (Toll-like receptor agonists; vaccine adjuvants) [125], 5-fluorouracyl (anti-cancer agent, antimetabolite) [126], and irinotecan (anticancer agent, topoisomerase 1 inhibitor) [117]. Recently, cubososme nanoparticles have been shown to potentiate the adjuvant properties of immunostimulants, and are thereby expected to stand in a brighter spotlight for the development vaccine formulations [127]. It is worth noting that liquid crystalline nanostructures are relatively novel phenomena and are likely to be subjected to further extensive research in the realm of controlled release parenteral delivery systems.

#### 3.1.2. Systemically Injectable Long-Acting Nanoparticle-Based Formulations

In general, intravenous injection is not an appropriate route for the prolonged delivery of naked drug molecules. Nonetheless, nanocarriers have provided exciting opportunities for the development of long-acting systemically injectable formulations. It goes without saying that an extended release of the drug from systemically injected nanocarriers necessitates their prolonged presence in the systemic circulation. As the concept of long-acting and systemically targeted nanoparticles have been often inseparably investigated, more detailed information regarding the formulation of such particles will be presented under the section related to the latter. It is, however, worth noting that long systemic circulation can facilitate a prolonged release of the payload from the carriers. Alternatively, a sustained release of the drug can occur following the accumulation of the circulating nanoparticles within the target tissue. For such reasons, systemic long acting and passively targeted formulation often go hand in hand, though few studies have focused on the development of nanosized systems with the sole aim of prolonged drug delivery. Examples include the PLGA-PEG nanoparticles of low molecular weight heparin (for the treatment of venous thrombosis) [128] and bovine serum albumin (a model protein) [129], PEGylated factor VIII (for treating hemophilia) [130], and albumin-conjugated peptide HIV (human immunodeficiency virus) fusion inhibitor (anti-AIDS) [131].

### 3.2. Nanoparticles for Tuning the Onset of Drug Release

**Figure 3:**
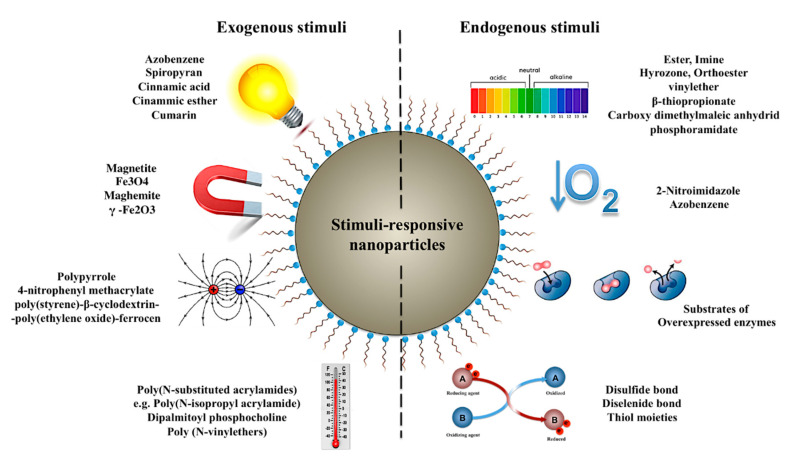
Various endogenous and exogenous stimuli exploitable for the design of stimuli-responsive nanocarriers along with the materials, moieties and cross-linkers sensitive thereto.

One desirable characteristic of an ideal delivery system is to release the incorporated payload at the right time and in the right place, so that the adverse effects of the drug on non-target organs or tissues as well as the required dosage can be alleviated. A potential contribution of nanotechnology to achieve the abovementioned objective is the design and development of delivery systems capable of releasing the incorporated therapeutic cargo in response to specific internal or external stimuli [132]. The significant allure of stimuli-responsive nanocarriers lays in the treatment of conditions, wherein the drug release can be initiated in response to the pathological triggers unique to the diseased organ or tissue [133]. Nonetheless, extrinsically induced onset of the drug release following the accumulation of the nanocarriers in the destination organ has provided exciting opportunities for the treatment of several disorders including cancer [134]. Literature holds different classifications of stimuli-responsive nanocarriers based on the origin (exogenous vs. endogenous) or the nature (environmental, biochemical, physical and chemical) of the triggering stimulus [135,136]. Herein, we present the most important of these stimuli, and review the approaches hitherto studied for the development of such stimuli-sensitive nanocarriers. A summary of these is represented in Figure 3.

#### 3.2.1. Temperature-Responsive Nanocarries

Temperature-sensitive nanocarriers have been designed with the main objective of releasing the incorporated payload in response to the internal or external changes of the ambient temperature. While externally induced hyperthermia is the major trigger of the drug release in such systems, internally elevated temperatures is observed in several disorders including infections, inflammation and cancer [135]. Thermosensitive nanocarriers comprise at least one component (polymeric or lipidic), which undergoes drastic physicochemical changes in response to the change of temperature. This temperature-sensitive material can be used as the main component for the fabrication of the naoparticulate systems or for the modification thereof [137].

Thermosensitive polymers such as poly(N-substituted acrylamides) [138] and poly (N-vinylethers) [139] undergo a reversible sol-gel transition in response to the shift of the temperature around their lower critical solution temperature (LCST). These polymers are soluble when exposed to temperatures below their LCST. Under such conditions, a swollen state is observed in the polymer medium, which corresponds to the formation of hydrogen bonds between the water molecules and the functional groups of the polymer structure. As the temperature surpasses the LCST of the polymer, however, the structure collapses due to the hydrophilic-hydrophobic transition. The resultant volumetric shrinkage banishes the incorporated drug molecules from the system [140]. A widely investigated example includes nanocarriers prepared or modified with Poly(N-isopropyl acrylamide) (PNIPAAm), a thermosensitive polymer with an LCST of about 32 °C. The attraction of PNIPAAm lies within the proximity of its LCST to the physiological temperature of the human body. A modification of the polymer’s LCST is possible with the help of additives (e.g., salts and surfactants) or through the structural incorporation of hydrophobic or hydrophilic monomers [141]. This would endow the polymer appropriate properties for the design of different temperature-sensitive nanocarriers, such as polymeric nanospheres [142] and micelles [143], as well as surface modified inorganic nanoparticles [144,145] and lipid-based nanostructures [146].

Another approach for the development of thermosensitive nanocarriers can be based upon the polymeric structures that swell, rather than shrink, above their so-called upper critical solution temperature (UCST). Nanogel systems prepared with acrylamide and acrylic acid exhibit such a behavior in the presence of sodium chloride or similar salts, where the swelling above UCST triggers the release of the entrapped cargo [147]. Compared with the LCST-based polymeric nanoparticles, however, these systems have been less widely investigated.

Apart from modification with thermosresponsive polymers, temperature-responsive lipid-based nanostructures such as liposomes can be also prepared using thermally sensitive lipids such as dipalmitoyl phosphocholine (DPPC), which possesses a phase transition temperature of about 41–42 °C. Above this temperature, the lipid will undergo a gel to liquid crystalline phase transition, facilitating thereby the release of the loaded cargo [148]. The composition of the thermosensitive liposomes can be manipulated to enhance their properties. For instance, to improve the rate of the drug release upon stimulation, and to reduce the associated phase transition temperature, lysolipid monopalmitoyl phosphocholine (MPPS) has been incorporated into the liposome structure [149]. An alternative strategy for the development of thermosensitive liposomes is the incorporation of poloxamers within the liposome formulation. As the temperature moves beyond the critical micellar temperature of these surfactants, partitioning of poloxamers into the phospholipid bilayer disrupts the liposomal structure and triggers the release of the payload [150]. Thermosensitive nanocarriers have been of particular interest in cancer therapy, where they have been investigated for the delivery and site-specific release of different chemotherapeutic agents, such as 5-fluorouracyl [117], paclitaxel [151], gemcitabine, and oxaliplatin [152,153], SN-38 [142], C6 (a permeable analog of ceramide, pro-apoptotic) [154], etc.

#### 3.2.2. Light-Responsive Nanocarriers

Light is a convenient release trigger given a variety of advantages it offers. These include non-invasiveness, spatial and temporal controllability, diversity of the applicable spectrum and the inducible photochemical reactions, and possibility of remote handling [135,155].

A variety of strategies have been developed to render nanomaterials light responsive, the detailed discussion of which is out of the scope of this review. Herein, we seek to merely present an overview of the most important of these approaches. For more detailed information, the reader is referred to a comprehensive review by Fomina et al. [156]. One common strategy to endow nanocarriers with visible or ultraviolet (UV) sensitivity is the incorporation of materials susceptible to photochemical reactions such as photoisomerization, photocrosslinkage and photosensitization-induced oxidation [157]. Photoisomerization-induced drug release occurs as a result of the structural disturbance of the carrier system due to the light-induced conformational changes around a bond with rotation restrictions (e.g., a double bond) [156]. The most commonly investigated materials with such properties include the UV-sensitive azobenzene and spiropyran [158,159]. Similarly, photo-induced cross-linkage of nanoparticles can cause structural disconformity, triggering drug release from the nanoparticles prepared or modified with UV sensitive materials such as cinnamic acid, cinammic ester and cumarin [158]. Photosensitization-induced oxidation is of particular interest for the preparation of light-responsive liposomes, where the formation of singlet oxygen following the illumination of a sensitive molecule can lead to the disruption of the lipid bilayer as a result of phospholipid oxidation [156]. This mechanism has been also exploited to enable a light-induced endosomal escape of the nanoparticles following internalization [160]. Further approaches to induce UV-visible responsiveness include photochemical hydrophobicity switch in micellar formulations [161], photo de-cross-linkage of sensitive copolymers [162], incorporation of gold nanoparticles in liposomal formulations [163], and photo-induced charge reversal [164].

A major limitation of the UV and visible spectra is their limited penetration depth (about 10 mm) in the body. The application of these is thus limited to superficially (e.g., subcutaneously or intradermally) accumulated nanoparticles [135]. Also, the potential harmful impacts of the UV light on the healthy cells and tissues should not be underestimated [165]. As a superior alternative to UV and visible lights, the near infrared (NIR) spectrum is associated with benefits such as higher biological friendliness, deeper tissue penetration and lower scattering characteristics [135,166]. Multiple mechanisms have been proposed to benefit from the NIR spectrum as a drug release trigger. For instance, photosensitive materials capable of absorbing two photons of NIR can sometimes initiate the same photochemical reactions induced by the UV/visible spectra [155]. Alternatively, nanosystems can be fabricated using NIR-to-UV/visible upconverting materials [167]. Finally, photothermal conversion of NIR radiation using size-specified gold nanorods has been proposed to release the drug from thermosensitive nanocarriers [168].

#### 3.2.3. Hypoxia-Responsive Nanocarriers

Hypoxia, the state of inadequate oxygen availability, is a hallmark of various disorders such as cancer, ischemia, rheumatoid arthritis, cardiomyopathy and vascular diseases [169]. In cancerous tissues in particular, the rapid cellular proliferation, exponential growth and faulty microcirculation create a hypoxic gradient, with the oxygen levels approaching values of 0–0.25 mm Hg within the deep tumor tissue [170]. Hypoxia can account for other unique abnormalities in the cancerous tissue, including the acidic and reductive nature of the tumor microenvironment, whose exploitation can open door to further possibilities for stimuli-responsive drug delivery, and which will be separately discussed under the corresponding sections [169].

Despite its association with a poor prognosis and its encouraging role in the tumor development and chemoresistance, hypoxia can serve as a specific tumor-induced trigger ensuring the site-specific release of the drug molecules from nanocarriers [171]. The majority of hypoxia-sensitive nanocarriers have been modified with hypoxia-responsive moieties such as 2-nitroimidazoles [169] and azobenzene [170,172,173,174]. For instance, under normoxic conditions, 2-nitroimidazole is oxidized back to its initial state following intracellular reduction [175]. Hypoxic conditions, on the other hand, lead to the conversion of 2-nitroimidazole to 2-aminoimidazole, which initiates the cargo’s release by disrupting the structure of the nanocarrier to which the moiety is grafted [176]. Similar consequences follow the reduction of the azobenzene group under hypoxic conditions [173]. Hence, modification of different types of nanocarriers with the aforementioned moieties can enable a hypoxia-induced trigger of the drug release, and is thus advantageous for site-specific drug release in cancer, cardiovascular disorders, and rheumatoid arthritis.

#### 3.2.4. pH-Responsive Nanocarriers

Development of pH-responsive nanocarriers has been pursued for the purpose of initiating the drug release within the organs, tissues and intracellular compartments with pH values deviating from the physiological norms [177]. pH-Responsive drug delivery at the organ level is mostly limited to the gastrointestinal tract and is often fulfilled using the orally administered dosage forms. Consequently, the focus of the current section will be mainly upon the pH-sensitive nanocarriers triggering the release of the drug under the acidic pH of certain tissues and organelles.

One of the most typical tissues of acidic nature is the tumor extracellular environment, whose slightly acidic pH (between 6.5–7.2) is a byproduct of enhanced lactic acid production and retention under the intratumoral hypoxic conditions [178]. This unique property of the tumor microenvironment has been, on the one hand, taken advantage of to enable an onsite release of the anticancer cargos within the tumor extracellular environment [179,180]. On the other hand, the acidic microenvironment has been exploited to remove the hydrophilic stealth coating of the particles and to exhibit the underlying cationic surface, which increases the internalization of the particles by enhancing their interaction with the negatively charged membrane of the cancer cells [181,182].

Intracellular compartments such as endosomes and lysosomes undergo rapid acidification, mainly owing to a vacuolar ATPase-mediated proton influx. Following the endocytosis of foreign particles or materials, a reduction of the pH to 5.0–6.5 and 4.0–5.0 is observed within the endosomes and lysosomes, respectively [183]. This acidification of lysosomal and endosomal compartments provides exciting opportunities for a pH-triggered site-specific drug release following the cellular internalization of the nanocarriers. Accordingly, significant effort has been dedicated to the development of nanocarriers with the potential to fulfill the abovementioned goal, the majority of which serve the intratumoral release of anticancer drugs [184,185].

Despite the diversity of the materials used for the fabrication of pH-responsive nanocarriers, the function of these systems is based on two general strategies. One mechanism involves the preparation of the nanocarriers using materials with ionizable functional groups, which due to protonation in acidic pH trigger drug release following the disruption of the nanocarrier structure [186]. The second approach is based on the cleavage of acid labile bonds within the nanoparticle structure, between the drug and the polymer, or between the nanoparticle and the stealth coating [186]. The most common pH-labile cross-linkers include the ester, hyrozone, carboxy dimethylmaleic anhydride, orthoester, imine, β-thiopropionate, vinylether and phosphoramidate [177]. Table 3 presents examples of the numerous hitherto-explored pH-responsive nanocarriers with potential for parenteral delivery.

#### 3.2.5. Redox-Responsive Nanocarriers

Redox-responsive nanocarriers often comprise chemical groups sensitive to oxidation or reduction [198]. Among these, nanocarriers susceptible to reduction are paramount, particularly for triggering the intracellular release of the drug cargos, nucleic acids and proteins [199,200]. The function of these systems is based on the substantially higher intracellular glutathione (GSH) concentration compared to that of the extracellular environment (about 10 mM vs. 2 µM, respectively) [201]. The most common approach to endow a nanocarrier redox-responsiveness is through the incorporation of GSH-responsive cross-linkers with disulfide bonds within the particle structure, between the particle and the stealth coating or the particle and the drug payload [202,203,204]. Thiolated nanostructures can respond to the higher intracellular GSH concentrations in a similar manner [205]. Alternatively, diselenide containing polymers have been synthesized and used for the fabrication of selenium-based redox-responsive nanocarriers [206].

A further application of redox-responsive nanosystems is to increase the specificity of the drug release locus to the cytosolic compartment of the tumor cells, where the GSH concentration is at least four times higher than normal [207]. Of particular interest in cancer therapy have been the GSH-sensitive polymeric nanoparticles with disulfide linkage [208]. These carriers have even been shown to help overcome multidrug resistance (MDR) in cancer cells, which is partially attributable to the higher intracellular GSH concentration within the resistant tumor cells compared to their non-resistant counterparts [209]. As an alternative to the use of redox-sensitive nanocarriers, the incorporation of a GSH-sensitive payloads such as redox-responsive pro-drugs have been exploited to enable a cancer cell specific intracellular trigger of the chemotherapeutics such as cisplatin [210]. In general, a combination of different targeting strategies and redox responsiveness seems promising to facilitate a site-specific intratumoral release of the chemotherapeutic cargos.

#### 3.2.6. Enzyme-Responsive Nanocarriers

Substrate-incorporated nanocarriers provide further opportunities for the site-specific release of the APIs. Upon the biocatalytic action of the enzyme on the corresponding substrate, a programmable onset of drug activation or release can be achieved at the desirable location [211]. Application platforms for enzyme-responsive nanocarriers in drug delivery are ample. The particles might be developed to release the drug in response to the abundance of certain enzymes in specific organelles, cells, tissues or organs, or dysregulated enzymatic activity under a variety of pathological conditions such as cancer and myocardial infarction [212,213]. Furthermore, the incorporation of substrates specific to bacterial enzymes can trigger the release of the drug cargos, specifically after the carrier uptake by microbial invaders [214]. Enzyme-responsive nanocarriers have been also developed to improve the outcome of nanoparticle-based gene delivery [215].

In general, two main classes of enzymes have been exploited for the formulation of such systems; hydrolases and oxidoreductases. The former comprises different subcategories such as proteases, lipases, and glycosidases, which act upon peptides, lipids and carbohydrates, respectively [211]. Among such substrates, peptides have been of particular interest due to formulation convenience and the established dysregulated activity of certain proteases in various disorders. Examples include capthesins (cancer, atherosclerosis, osteoporosis, Alzheimer’s disease), kallikreins (cancer, hypertension and inflammation), serine proteases (cancer), caspases (neurodegenerative disorders), matrix metalloporinases (MMPs; cancer, bronchiectasis, chronic asthma, cystic fibrosis, chronic obstructive pulmonary disease (COPD), etc.), and disintegrin and metalloproteinase domain protease (ADAM; Alzheimer’s disease) [216].

Substrates can either serve as enzyme-cleavable cross-linkers between the drug and nanoparticle [217,218], or else form the enzyme-sensitive building blocks of the nanocarrier structure. An example of the latter involves the use of protein/polysaccharides for the development of supramolecular assemblies, which trigger micellar dissociation upon exposure to proteases/glycosidases [219]. Furthermore, enzyme-responsive moieties can serve as gatekeepers that control the onset of drug release from mesoporous silica nanoparticles (MSNs) [220,221]. Enzyme-responsive substrates have been also utilized for the fabrication of pro-drugs, rendering them inactive until subjected to enzymatic biocatalysis in the destination tissue [216]. Yet, a further application of enzyme-responsive substrates is the on-demand shedding of the stabilizing polymers and stealth coatings [211]. For instance, enzyme-responsive cross-linkers can be used to develop highly stable polymer-caged lipososmes, which manifest the properties of the original liposomal formulation upon the biocatalytic shedding of the caging polymer. This, in case of the low stability of the caged lipososmes, can also lead to a spontaneous and on-command release of the drug cargo at the desirable locus [222].

#### 3.2.7. Electroresponsive Nanocarriers

Weak electric fields (usually below 1 V) can serve as attractive exogenous stimuli to trigger drug release from the delivery systems responsive thereto. This attraction mainly lays within advantages such as ease of generation and high controllability along with the possibility of remote application and simplicity of the required equipment [223]. However, given the low tissue penetration depth of such electric fields, application of electroresponsive systems is often limited to superficial tissues [135]. Hence, it is no surprise that many of such systems have been developed as implantable devices of various shapes and sizes. Nonetheless, electroresponsive nanocarriers are of particular interest for two main reasons. Firstly, as injectable systems, their administration is associated with lower invasiveness than the surgical procedures necessary for the application of macrosized implants. Second, their large surface area to volume ratio allows for significantly higher drug loading [223]. To develop implantable electroresponsive nanoparticles, Ge et al. dispersed polypyrrole nanoparticles within a thermosensitive in situ forming hydrogel [224]. Being developed from a conductive polymer, the embedded polypyrrole nanoparticles release their drug content once the implant is exposed to an appropriate external electric field. A further example includes the use of electroresponsive units 4-nitrophenyl methacrylate (NPMA) for the development of macromolecular units coating the surface of MSNs. Upon the application of an external electric field, the conformation of NPMA monomers will be reoriented, which triggers the release of the encapsulated API [225]. Adjunct to the abovementioned examples, electric fields have been also exploited to enable the self-assembly and disassembly of elcetroresponsive block copolymers of poly(styrene)-β-cyclodextrin-poly(ethylene oxide)-ferrocene [226].

Conventional use of exogenous electrical fields to the contrary, abnormal electrical activities associated with seizures has been exploited as an endogenous stimulus to trigger the release of antiepileptic drugs upon demand. Wang et al. developed phenytoin sodium loaded electroresponsive nanogels modified with the brain targeting peptide angiopep-2 [227]. Depending on their content of sodium 4-vinylbenzene sulfonate, different degrees of electrorespnsiveness was observed both in vitro and in vivo, triggering thereby the release of the incorporated antiepileptic drug phenytoin sodium under the effect of generalized tonic-clonic seizures [227,228]. Such a system can thereby shift the application of electroresponsive nanocarriers to beyond the conventional platform and depicts new and exciting horizons for the treatment of seizure and epileptic disorders.

#### 3.2.8. Magnetically Responsive Nanocarriers

Given their remote controllability and intrinsic tissue penetrability, applications of magnetic fields in drug delivery and diagnostics are abundant. Magnetic fields can be exploited as guides for targeted drug delivery and diagnostics, for the induction of local hyperthermia, for magnetic resonance imaging, and for on-command release of the therapeutic cargo from delivery systems [135,229,230]. While in most cases an amalgamation of these effects is desired, our focus here will be solely upon their potentials for programmed drug release.

Structurally speaking, magnetic nanoparticles can comprise of a magnetic core (e.g., magnetite, Fe_3_O_4_, or maghemite, γ-Fe_2_O_3_) with a functionalizable polymer or metal coating, or else a porous polymeric matrix with intraporous-precipitated magnetic nanoparticles [229]. These can be prepared through a variety of strategies, such as wet precipitation or co-precipitation, reverse micelle mechanism, chemical vapor condensation, and lipid phase reduction, which have been elegantly reviewed elsewhere [231]. Magnetic fields can enable an on-demand onset of drug release based on two different mechanisms. Either the on-site trigger of drug release occurs due to magnetically induced direct structural rearrangements within the responsive nanocarriers, or else is a byproduct of the hyperthermic effect of the magnetic field [232]. An example of the former includes an in situ forming ferrogel composed of magnetic iron oxide nanoparticles and pluronic-F127 micelles incorporating indomethacin as a model hydrophobic drug. As the iron oxide particles advance toward each other when exposed to the appropriate magnetic field, the micelles are squeezed, banishing thereby the drug molecules from their hydrophobic cavities [233]. The majority of the magnetically responsive nanocarriers, however, benefit from the hyperthermic effect of exogenous magnetic fields. The spontaneous release of the drug in this case can either pertain to the breakage of thermosensitive bonds between the drug and the nanoparticle, or else the increased permeability of the carrier due to structural damage or nanopore formation [234]. Magnetoliposomes are one of the earliest examples within the context of the latter, where an increase of the bilayer permeability can be achieved by magnetic heating of the system close to the membrane melting temperature [235]. Additionally, instantaneous drug release following the exposure of iron oxide encapsulated porous silica nanocapsules [236], iron oxide embedded Pluronic-F127 nanosphers [237], iron oxide capped mesoporous silica nano-rods [238], iron oxide-DNA gated MSNs [239], folic acid and cyclodextrin-functionalized supramagnetic iron oxide nanoparticles [240], etc. occurs through similar mechanisms. Given their multiple applications in diagnostics, drug targeting and controlled delivery, magnetic nanoparticles are expected to be the subject of further extensive research in the future.

#### 3.2.9. Dual and Multi-Stimuli-Responsive Nanocarriers

Dual and multiple stimuli responsive nanocarriers have been developed to fulfill different, and rather diverse, objectives, and can hence offer a verity of benefits. First, exploitation of multiple stimuli associated with a specific disorder, e.g., abnormal pH, dysregulated enzymatic activity, and hypoxia in case of cancer, can enable a more selective release of the drug at the diseased tissue [241]. Second, several stimuli can be combined to help accomplish various stages of the nanocarrier mission. For instance, while one stimulus can be exploited to shed the stealth coating of the nanoparticles, others can serve to trigger an on-demand drug release within the target site or inside the desired cells [242]. Third, given the patient-to-patient differences in term of endogenous stimuli, their complementation with an exogenous trigger can significantly enhance controllability. And finally, certain exogenous stimuli such as magnetic fields are often included due to their targeting and guidance potentials.

A review of the literature reveals the research on the development of multi-stimuli responsive nanocarriers to have remarkably grown in recent years. In particular, efforts have been made to design highly controllable nanocarriers that respond to a larger number of stimuli. Dual magnetic and pH responsive chitosan nanoparticles [243], triple temperature, pH and redox responsive assemblies of tetrahydropyran-protected 2-hydroxyethyl methacrylate and PANIPAM copolymers [244], and quadruple temperature, pH, light, and redox responsive nanoassemblies of amphiphilic diblock copolymer poly(2-nitrobenzyl methacrylate)-SS-poly(dimethylaminoethyl methacrylate) [245] are only a few examples of multi-stimuli responsive systems developed within the recent years. It should be noted, however, that notwithstanding the unique advantages offered by such systems, their complex nature is a major drawback complicating their clinical translation [135]. Moreover, given their relatively large size (often above 200 nm), investigation of their biodistribution and intracorporeal fate is warranted [246]. Finally, such nanocarriers have often limited or no biodegradability, which needs to be further addressed for improved clinical application [246].

## 4. Targeted Drug Delivery

Of all unique applications of nanoparticles for parenteral administration, targeted drug delivery is to this day the most extensively explored. The horizons of nanoparticle-mediated targeting was depicted over a hundred years ago, as Paul Ehrlich proposed his well-known “magic bullet concept” about the drugs that could go straight to their intended cellular and intracellular targets [247]. The discovery of the enhanced permeability and retention (EPR) effect in 1986 provided a more solid platform for the realization of this concept, giving birth, in less than a decade, to Doxil, the first FDA approved intravenously injectable liposomal formulation for the targeted delivery of doxorubicin to solid tumors [248]. Today, a myriad of nanoparticulate systems are under development and investigation for targeted drug delivery in the treatment of disorders beyond cancer, with many having already opened their way to the pharmaceutical market, and more still going through various stages of clinical trials (see Table 1 and Table 2) [8,249]. The reason for such a rapid growth majorly lies within the benefits of enhancing the ratio of on-target to off-target accumulated drug molecules. Particularly for those drugs which are potent or exert their effect indiscriminately all over the body, such an increase can both significantly improve the therapeutic efficiency and reduce the undesirable side effect in the clinic, increasing thereby the patient’s benefit [250]. As the application of nanoparticles for drug targeting is extensive, and has been abundantly debated in numerous review articles and perspectives, we will only present a brief discussion of the principles of nanoparticle-based drug targeting to various healthy or diseased organs, tissues, cells, and intracellular compartments following injection-based routes, and in particular intravenous administration.

## 5. Passive Targeting

Passive targeting is often the byproduct of the nanoparticle potentials to accumulate in certain tissues, organs or cells. Although efficient nanoparticle design is imperative to meet the specific requirements of passive accumulation in the organ of interest, this is still more a function of the particles’ physicochemical properties and the unique characteristics of the target site, rather than functionalization with certain targeting moieties which underlies the active targeting approaches. The concept of passive targeting is highly perceived in case of cancer therapy, where, in comparison to the free drug molecules, nanocarriers tend to accumulate more efficiently in the tumor microenvironment by virtue of the EPR effect. Notwithstanding such a perception, passive targeting can stretch well beyond drug delivery to the tumor microenvironment, with potential exploitation for selective drug delivery to various targets of interest.

Prior to elaboration on the concept of nanoparticle-mediated passive targeting, it is worth to mention that in some cases, the passive deposition or penetration of the nanocarriers in the target of interest can be externally promoted. Examples include the use of ultrasound, radiation, hyperthermia, and photochemical tissue penetration for enhancing the passage of the nanocarriers in the tumor microenvironment or across the BBB [251,252]. These methods have hitherto yielded promising results and are expected to provide a more commonly used clinical platform within this context.

**Figure 4:**
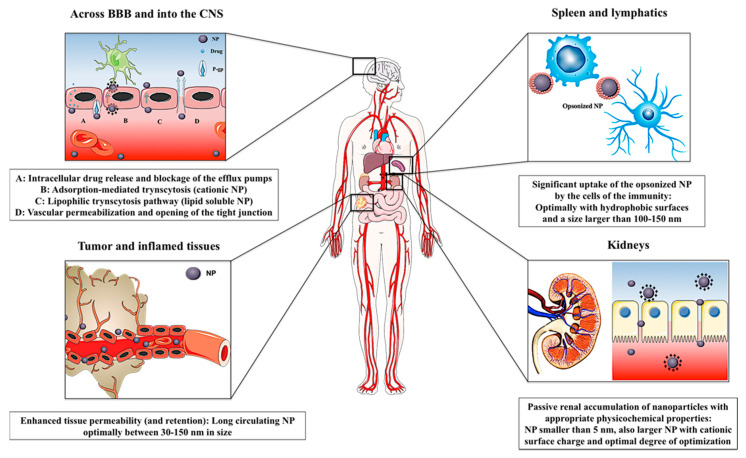
Potential targets for the passive accumulation of nanoparticles following intravenous injection.

As previously debated, a long circulation time is a prerequisite for the passive accumulation of the drug in the target of interest. To this end, nanoparticulate carriers should possess “stealth” properties, in other words to be capable of evading clearance by the kidneys and the mononuclear phagocyte system (MPS), which is also known as reticuloendothelial system [253]. The renal clearance of the particles is mostly a function of particle size, where nanocarriers smaller than the renal fenestration (maximum size 20–30 nm) are often excreted by the kidneys [254]. Particles larger than 100 nm often fall victim to clearance by MPS, which comprises mononuclear phagocytic cells stationed in liver and spleen. These phagocytic cells are essentially responsible for the removal of small exogenous particles from the systemic circulation [255]. Therefore, carriers whose hydrodynamic diameter lies within the range of 30–100 nm can evade both the renal excretion and MPS clearance, and thereby possess inherent long circulating properties [254]. For larger nanoparticles, recognition by MPS requires pre-labeling by serum proteins, a process referred to as opsonization [256]. Susceptibility to opsonization is determined by the surface charge and hydrophobicity of the nanoparticulate systems [257]. Even though hydrophobic and charged nanocarriers are more prone to absorb opsonins, they can be superficially modified to grow less MPS-attractive. The most common approach is grafting hydrophilic polymers, such as PEG, polysaccharides, poloxamers, and poloxamines, to the nanoparticle surface [254]. Interestingly, it seems that surface modification with hydrophilic moieties such as PEG does not reduce the opsonization process per se, but changes the composition of the adsorbed protein corona. While albumin, vibronectin and fibrinogen often form the main corona around non-pegylated particles, their pegylated counterparts seem to get marked by clusterin, which is believed to be responsible for the lower MPS attractiveness of such stealth nanoparticles [258]. Alternative strategies for increasing nanoparticles’ circulation time include surface modification with CD47 “self peptides”, surface coating with leukocyte and erythrocyte membranes, and suppression of the MPS by means of different inhibitors [259,260,261]. For protein and peptide drugs, an alternative approach to develop long circulating formulations includes chemical modifications of the molecules by PEGylation, hyperglycosylation and mannosylation [94]. Conjugation of the drug molecules with natural proteins such as albumin [262] and with fatty acids [263] can also enable the development of long circulating formulations. In the following, the concept of passive targeting to various tissues/organs or in various disorders will be briefly debated (Figure 4).

### 5.1. Inflammatory Disorders

A particular niche of nanoparticle-based drug delivery is revealed in the targeting of inflamed tissues with abnormal immune cell infiltration. The most renowned example is of course the tumor microenvironment, where the presence of numerous angiogenic blood vessels with defective and leaky vascular structures along with the low intratumoral lymphatic drainage prompts the intratumoral deposition of nanoparticles with appropriate sizes (generally smaller than 400 nm, optimal size is about 10–200 nm) [264]. Despite the absence of low lymphatic drainage, other inflamed tissues are also associated with enhanced vascular leakage due to the contraction of the endothelial cells that line the arterioles and capillaries under the abundance of various inflammatory mediators such as bradykinin, histamine, leukotriene, etc. [265]. Such unique characteristics facilitate the nanoparticle-mediated passive targeting of various systemic inflammatory disorders, including rheumatoid arthritis [266] and systemic lupus erythematosus [267]. Interestingly, the EPR phenomenon has been also reported in case of other pathologies, such as infections and myocardial infarction [268], which provide further platforms to expand the application of nanoparticles for passive targeting purposes [259].

### 5.2. Central Nervous System (CNS)

Relative to free drug molecules, nanoparticles can also facilitate drug targeting to the CNS, which in particular paves the way for the treatment of various neurodegenerative disorders [269]. The key role of many nanocarriers within this context lies within the ability of long-circulating nanoparticles to circumvent the blood brain barrier (BBB). While active targeting approaches can enhance the CNS targetability of these systems, many different types of nanocarriers can improve the transport of the drugs across the BBB in a passive manner, increasing thereby their accumulation in the CNS. Depending on the composition and physicochemical properties of the particles, they can improve the BBB penetrability through several different mechanisms. The most basic mechanism involves the permeabilization of the capillaries and opening of the tight junctions [270,271]. Transcytosis (e.g., through adsorption-mediated or lipophilic pathways) is a further potential approach through which nanoparticles with appropriate physicochemical properties (e.g., cationic or lipid-soluble nanoparticles) can overcome the BBB even in the absence of any active targeting moiety [272]. Alternatively, many nanoparticles are taken up by the endothelial cells of the capillaries, releasing their contents within the cytoplasm of such cells, where they will be subsequently exocytosed to the abluminal side [273]. Finally, the presence of various amphiphilic molecules in the structure of nanocarriers (used for stabilization and stealth coating) endows them inherent abilities to inhibit the efflux pumps such as P-glycoprotein (P-gp), which will in turn increase the drug transport across the BBB [274,275]. All these can act in favor of a superior passive CNS deposition of the drug molecules when loaded within long-circulating nanoscale carriers.

### 5.3. Kidneys

A further, yet less frequently debated, potential destination for passively targeted nanoparticles is the kidneys. Though kidney is one of the organs responsible for the clearance of nanoparticles of small sizes, the substantial part of any injected nanoparticle dosage will end up in the liver and the spleen. Nonetheless, nanocarriers can be designed to provide a decent concentration of the incorporated drug in various parts of the renal tissue, which, in addition to diagnostic purposes, is indeed exploitable for the treatment of various renal or even systemic disorders including acute kidney injury, chronic kidney disease, glomerular diseases, kidney cancer and hypertension [276]. The ability of nanoparticles to passively and predominantly accumulate in kidneys seems to be dependent upon their physicochemical properties and degree of opsinization. For instance, long circulating “mesoscale” PEG decorated PLGA nanoparticles of about 400 nm have been reported to deposit in the kidneys 7 times more efficiently than in other organs [277,278]. Nanoparticle size can be further manipulated to enable a more specific targeting of various renal compartments. In general, super small particles of about 5 nm have the potential to pass through the glomerular filtration barrier, which enables their subsequent absorption by the epithelial cells lining the renal tubule [279]. Adjunct to nanoparticle size, surface charge seems to be an important determinant of nanoparticles’ renal deposition. While nanoparticle opsonization and the formation of a biomolecule corona often result in a deviation from the original nanoparticle charge following intravenous injection, maintaining a cationic surface charge has been reported to favor the passive glomerular accumulation of iron oxide nanoparticles [280]. Hence, to enable the passive renal deposition of nanoparticles, optimization of their surface and physicochemical properties is warranted.

### 5.4. Spleen and Lymphatics

In contrast to the above-mentioned scenarios, a long circulation time is not a prerequisite for the passive targeting of organs such as spleen. Splenic accumulation of nanoparticles is of particular interest for the passive targeting of the immunotherapeutic payloads to the splenocytes, whose activation can exert substantial therapeutic effect in many disorders [281]. Furthermore, passive accumulation of the subcutaneously injected nanoparticles of 10–100 nm in the lymphatics provides a further platform for the optimal delivery of various vaccines and immunotherapeutics to their optimal site of action [282]. The lymphatic targeting of nanoparticles has been also used for purposes beyond vaccination and immunotherapy, for instance in the case of targeted drug delivery to lymphatic filarial parasites [283].

## 6. Active Targeting

**Figure 5:**
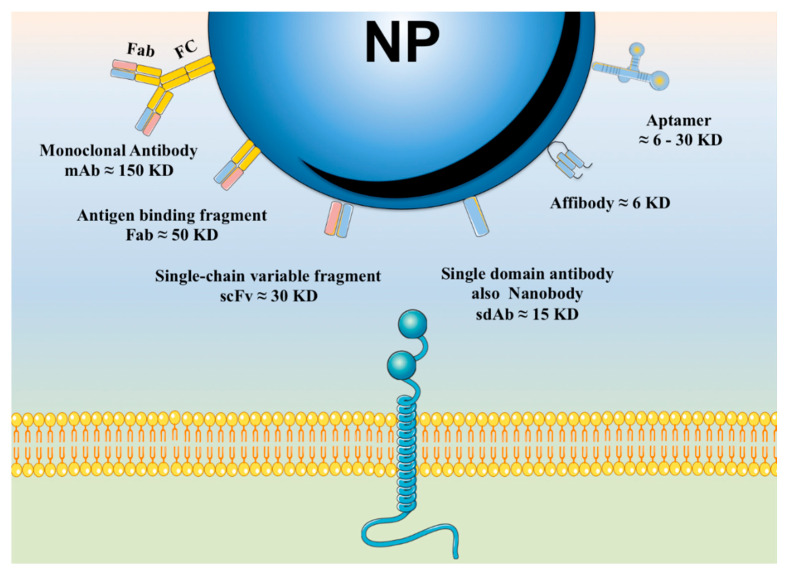
Presents a summary of some of the affinity molecules used for active targeting purposes.

Active targeting seeks to enhance the site-specificity of drug delivery through the modification of the nanocarrier surface with moieties that possess high affinity for certain molecules (receptors, enzymes, markers, antigens, etc.) abundantly expressed in the target of interest [284]. While active targeting mainly enhances the internalization of the nanocarrier by the cells of interest, it does not influence the biodistribution of the nanocarriers per se [285]. However, when combined with passive targeting approaches, active targeting can better reduce the undesired nonspecific interactions, thereby increasing the ratio of on-target to off-target drug molecules. Even though active targeting has been most extensively explored to enhance the delivery of chemotherapeutics to cancerous cells in the tumor microenvironment, the concept can be still applied for drug targeting to any healthy or diseased tissue, cell, or intracellular compartment with unique targetable features. Hence, unlike the section on passive targeting, our discussion here will revolve around the nature of targeting moieties used in nanoparticle design (Figure 5), rather than the potential targetable destinations.

### 6.1. Active Targeting Based on Affinity Molecules

#### 6.1.1. Affinity Proteins and Peptides

Affinity proteins possess a high selectivity in binding to certain molecular structures and are hence of great interest in active targeting approaches. Among various affinity molecules, antibodies are best known for their specific recognition of the antigenic epitope against which they have been developed. Structurally speaking, antibodies are Y-shaped glycoproteins comprising two antigen recognition domains (Fab fragment) and two identical domains with effector function (Fc fragment). Each Fab domain is in turn composed of a light (L: 24–25 kD) and a heavy (H: 55–70 kD) chain held together by disulphur bridges. Each chain has a constant (C_H_ and C_L_) and a variable (V_H_ and V_L_) segment, with the latter being responsible for antigen recognition function [286]. While the wealth of information in terms of production and modification often favors the use of full antibodies for targeting purposes, limitations such as immunogenicity, low stability, rapid elimination, and less than expected efficiency have promoted the generation and use of various antibody fragments [287]. Within this context, Fab type fragments [288], single chain variable fragments (scfV) [289], half-antibodies (hAB) [290], diabodies [291], and bispecific antibodies [292] have been tagged on nanoparticle surface to enhance their specificity for a certain target. Antibody fragments are advantageous over full immunoglobulins in terms of their lower immunogenicity, higher nanoparticle loading due to their smaller size, and better controllability of their orientation on the nanoparticle surface [287]. Depending on the nanoparticle structure and the possible chemical modification thereof, conjugation of full antibodies and their fragments can be achieved based on the formation of amide bonds, Schiff base linkage, hydrazone bonds, disulfide and thiolether linkages, as well as click reaction [293].

As an alternative to antibodies, other scaffold proteins can be used for active targeting purposes. These, also known as non-immunoglobulin scaffolds or antibody mimetics, reconcile the strong recognition ability of antibodies with further favorable characteristics including small size, robustness and high yield bacterial production [294]. Scaffold proteins are diverse and a detailed discussion of them all is out of the scope of this paper. Here, our focus will be mainly upon the protein scaffolds that have been reconciled with nanotechnology to achieve targeting in diagnostic and therapeutic arenas.

Affibodies, small (6 KD) affinity proteins (technically peptides) with a robust three-helix structure based on a modified B-domain of staphylococcal protein A, are the most widely known types of scaffold proteins [295]. Combinatorial randomization of the amino acids in 13 positions on helices one and two of the three helix bundle has provided a vast library of affibodies for 20 years [296]. Recently, affibodiy technology has been combined with nanotechnology to yield highly targeted nanoparticles both for diagnostic and therapeutic purposes. Most of such targeted nanoparticulate systems have been developed for the purpose of cancer management, and have benefited from the conjugation of affibodies against human epidermal growth factor receptor 2 (HER-2) to various types of nanoparticles [297,298] and liposomes [299].

A further category of protein scaffolds, affimers are derived from Adhiron scaffold, a synthetic thermally stable protein originally based on a cystatin consensus sequence, and is structurally related to a previously reported scaffold engineered from human stefin A [300]. Notwithstanding their relatively recent advent, these have been already exploited in nanocarrier development, not only to enable active targeting [301], but also for the purpose of nanoparticle morphology modification [302].

As our knowledge of the potential binding sites for various scaffold proteins increases, it is expected that these find a more distinct spotlight as active targeting moieties for nanoparticle modification. This is in particular evident from the fact that literature has lately witnessed the emergence of various scattered studies reporting the use of different types of scaffold proteins, such as chromobodies [303], Centyrins [304], DARPins [305], and repebody [306], for the nanoparticle-based active targeting of cancer.

In addition to antibodies and antibody mimetics, other peptides with high affinity for certain targets have been identified and exploited for nanoparticle modifications. Thanks to the development of peptide phage libraries, plasmid peptide libraries, bacterial peptide display libraries, and other novel screening technologies, numerous peptides have been identified and many have been successfully used to enable targeted nanoparticle-based drug delivery to different cells and even intracellular compartments [307]. A few examples include the cRGD peptide for targeting the tumor microenvironement [308,309,310], Angiopep-2 for targeting the BBB and CNS [311,312], kidney-targeting peptide (KTP) [313] and G3-C12 peptide [314] for renal targeting, and various nucleus localization signal (NLS) peptides for nucleus targeting [315]. Advantages such as small size, low immunogenicity, simple conjugation chemistry, acceptable stability, cost-effectiveneess, easy-to-scale-up production, and manipulation opportunities render peptides attractive targeting moieties in nanotechnology [316].

#### 6.1.2. Lectins

Lectin is a general term referring to proteins or glycoproteins with affinity for sugar moieties available in various glycoconjugates. Not only can lectins serve the purpose of site-specific nanoparticle-based drug targeting, they can also prolong the nanoparticle/drug residence in the target site, establish close contact between the nanoparticle/drug and the target cell membrane surface, and enhance the trans-epithelial drug transport rate through specific cellular interactions [317]. Even though several hundreds of lectins have been hitherto isolated from plant, invertebrate and animal sources over the years, the biological functions of these molecules along with the recognized carbohydrate sequences remain in many cases unclear [318]. This, however, does not suggest a lack of progress in the field of lectin engineering. On the contrary, numerous studies have been dedicated to the understanding of such interactions, and the subsequent exploitation of the knowledge for the increase of the lectin binding efficiency and specificity. Within this context, strategies based on protein, nucleic acid, and chemical engineering have been used. Nonetheless, their applicability to lectin engineering remains largely limited when compared to other functional proteins such as enzymes [319]. Furthermore, efforts have been made to develop small lectin-like peptides for drug targeting, as normal lectins with a molecular weight of more than 10 KD can result in toxicity and immunogenicity [320,321].

As potential binding sites for lectins require the superficial expression of a good number of sugar moieties, lectin targeting is often plausible for mucus secreting tissues such as the oral cavity [322], gastrointestinal tract [323], lungs [324], and corneal and conjunctival epithelia [325], as well as for nose to brain drug delivery [326]. Since these tissues are targeted using non-injectable formulations, a detailed discussion of these lies without the scope of the current paper and has been elegantly presented elsewhere [327]. Within the context of injection-based parenteral delivery, however, lectin-based targeting has been mainly exploited for tumor targeting. It is well established that malignant transformation in cancer cells leads to the aberrant expression of O-glycans as saccharide components of membrane-bound N-acetyl galactosamine (O-GalNAc) glycoproteins (T and Tn antigen), and glycolipids (Lewis a and Lewis x). These can serve as targets for various natural lectins [328]. To this date, lectin-targeted nanoparticles have been investigated for active drug targeting to various cancerous cells or tissues [329,330,331,332]. A detailed review of the current status of lectin-based targeting for cancer diagnosis and therapy is presented elsewhere [333]. As a bonus, some lectins have been reported to directly exert cytotoxic effects and hence can act as an anti-cancer actives as well [334].

#### 6.1.3. Glycans

The above-debated notion can be reversed to target endogenous lectins or carbohydrate receptors by decorating the nanoparticles with appropriate sugar or carbohydrate moieties, also known as glycosylation. Glycans can be classified both as affinity molecules and ligands for receptor targeting depending on the nature of the molecular target. Since the previous section dealt with the introduction of the lectin-based targeting, however, the glycan-based targeting will be elaborated here. Two strategies have been hitherto used for glycan-based targeting; the first involves the decoration of nanoparticles with various sugar moieties, among which glucosyl [335], mannosyl [336] and galactosyl [337] groups have been the most popular. A second strategy is based on the use of sugar-based polymers for nanoparticle preparation. Examples include polysaccharide derivatives, glycopolymers and sugar-linked polymers [338]. Regardless of the implemented strategy, glycosylated nanoparticles offer the advantage of possessing inherent stealth properties, while simultaneously serving the active targeting purposes [259]. There are several important targets for glycosylated nanoparticles. One is the Asialoglycoprotein receptor (ASGP-R) expressed exclusively by hepatic parenchymal cells, which enables targeted drug delivery to hepatocytes [339,340] and hepatocellular carcinoma [337,341]. Another potential target includes the glucose transporters (GLUTs), amongst which GLUT1 has been considered paramount. GLUT1 is a ubiquitous glucose transporter responsible for glucose uptake by erythrocytes and glucose transfer across the BBB, and is also overly expressed in tumors [342]. Accordingly, GLUT1-targeted glycosylated nanoparticles have been investigated for drug delivery across the BBB [343,344] as well as for active drug targeting to the brain cancers [345] and otherwise located tumors [346,347]. Yet another potential target for glycosylated nanoparticles is the C-type lectin receptors, mainly expressed by hepatic endothelial and Kupffer cells, macrophages and dendritic cells (DCs) [348]. The first type of these receptors is the mannose receptors, which have been exploited as a target for mannosylated nanoparticles for vaccine, antigen or adjuvant delivery to such antigen presenting cells (APCs) [336,349,350]. Other C-type lectin receptors such as DC-specific intercellular adhesion molecule-3-grabbing nonintegrin (DC-SIGN), langerin, human macrophage galactose- and N-acetylgalactosamine-specific C-type lectin (MGL), Dectin-1 or beta-glucan receptor, and DC immunoreceptor subfamily can also serve as targets for the delivery of immunomodulatory cargos using glycosylated nanoparticles [351]. Last but not least, the prevalence of lectin-like receptors and galactin receptors in the tumor microenvironment can justify the use of glycans as active targeting moieties for nanoparticle-based drug delivery in cancer [348,352,353].

#### 6.1.4. Aptamers

Aptamers are affinity molecules of oligonucleotide nature. They are single-stranded DNA or RNA oligonucleotides of 6–30 KD (usually 10–15 KD) with unique three-dimensional structures that endows them high affinity and specificity for certain target molecules. Aptamers are produced through SELEX (systematic evolution of ligand by exponential enrichment) technology. Despite the diversity of the available SELEX methods, they are all based on the repetition of four different steps: incubation of the target of interest with the available library of random aptamers, elution of the bound sequences, amplification of the bound sequences, and the separation of the single-stranded oligonucleotides [354].

To this day, numerous aptamers have been developed through SELEX technology and used to facilitate active targeting of various organic and inorganic nanoparticles to different types of cancer cells, [355,356,357] including drug resistant variants [358], as well as other cell types such as osteoblasts [359], immune cells [360] and many more. Adjunct to the conventional surface functionalization of nanoparticles, aptamers could be exploited as structural components of nucleic acid-based sequences that assemble to form three-dimensional nanostructures [358,361]. In general, aptamers offer advantages over conventional immunoglobulin affinity proteins. These include smaller size, higher stability to thermal, pH, and organic solvent-mediated degradation, and easier production [362]. Nonetheless, they are also associated with shortcomings including low serum stability due to sensitivity to nuclease degradation and high production costs [307]. The former has been addressed by introducing of several tweaks in their structure. These include capping the terminal ends of the molecule, substitution of the natural nucleotides for unnatural nuclease-unappealing alternatives, substitution of naturally occurring nucleotides with hydrocarbon linkers, generation of mirror image aptamers (spiegelmers), and reduction of conformational flexibility through locked nucleic acid modifications [363]. These have increased the feasibility of aptamers as active targeting moieties.

### 6.2. Active Targeting Based on Natural Ligand-Receptor Interactions

Nanoparticle decoration with the ligands of overly expressed receptors on the target of interest has provided a further popular platform for active targeting to various healthy or diseased destinations. Ligands used for targeting purposes need to have substantially high affinity and specificity for the receptor of interest, and relatively simple conjugation chemistry. For instance, abundant expression of transferrin receptors [364], folate receptors [365], lactoferrin receptors [366], low-density lipoprotein (LDL) receptors [367], interleukin receptors [368], somatostatin receptors [369], and lectin receptors (see under glycans) [329] on various potential targets such as the cancer cells, BBB, and inflammation sites have rendered their respective ligands attractive targeting moieties. Compared to affinity molecules, the lure of such natural ligands lies mainly within their prevalence, stability, inexpensiveness, and low immunogenicity. Even in some cases, targeting ligands such as LDL and high-density lipoprotein (HDL) have been directly formulated as nanoparticles wherein the drug molecules have been encapsulated [370,371].

## 7. Adjuvancy and Immune Activation

A less commonly debated domain to which injectable nanoparticles have assisted is the field of vaccination. The contribution of nanoparticles to this arena goes well beyond their ability to load, protect, target and deliver the immunotherapeutic cargos to the immune cells of interest, particularly APCs [372]. Those contributions certainly fall within the scope of the previously presented sections. Therefore, the focus of this section will be primarily upon the application of injectable nanoparticles as adjuvants and stimulators of the immune system. Needless to say, part of the immune response boosting potential of nanoparticulate vaccine carriers indeed originates from their ability to deliver a substantial number of antigens directly to the desired types of the APCs, thereby minimizing the amount of cargo that goes astray. Furthermore, nanocarriers can increase the visibility of molecular adjuvants to the cells of the innate immunity and thus increase their uptake thereby. This would also enhance the generated immune response compared to when adjuvants are used in a free form [373,374]. Nevertheless, the inherent immunostimulatory potentials of various nanoparticulate formulations have been already established [375]. Even though such intrinsic immunogenic properties have been more often debated from the immunotoxicological perspective, they have recently emerged as a novel platform to boost the efficacy of the available vaccination and immunotherapeutic systems.

It is widely known that the specific size range of nanomaterials promotes their recognition as “foreign” and “non-self” by the cells of the innate immunity [376]. Once on the loose in the systemic circulation, nanoparticles can get randomly tagged by various circulating antibodies, or else activate the complement system upon interaction with the blood opsonins. Both opsonization and antibody tagging make the particles prey to phagocytosis by macrophages via complement receptor-mediated and FC receptor-mediated pathways, respectively. Both pathways are known eventual triggers of the pro-inflammatory cascades [377]. A similar scenario occurs in case of the nanoparticles exhibiting superficial sugar moieties, for instance those decorated with mannose, which activate the inflammatory cascades upon uptake via macrophage mannose receptors [377,378].

**Figure 6:**
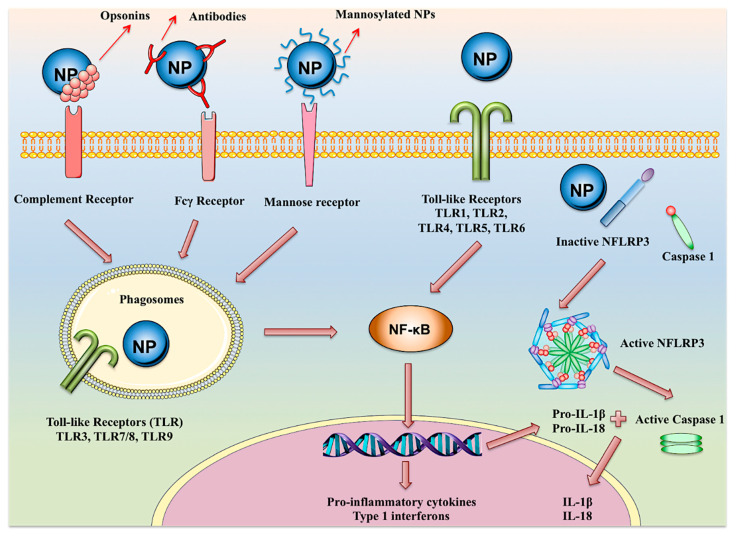
Summary of nanoparticle-mediated activation of pro-inflammatory pathways.

Alternatively, many nanoparticles have been shown to behave in a pathogen-mimicking manner, interacting with various pattern recognition receptors (PRRs). For instance, some nanoparticulate systems are believed to induce the self-oligomerization of Nod-like Receptor (NLR) family members and the subsequent activation of the inflammasomes (e.g., NLRP3), thereby triggering the autocleavage of caspase 1 and the subsequent production of IL-18 and IL-1β [376]. NLRP3 activation has been shown in case of a wide array of organic and inorganic nanoparticle such as morphous silica nanoparticles [379], silica dioxide and titanium dioxide nanoparticles [380], branched polyethylenimine and polyethylenimine-β-cyclodextrin nanoparticles [381], and many more. On the other had, a wide range of organic and inorganic nanoparticles have been shown to activate the immune response through direct interaction with various Toll-like Receptors (TLRs). Examples include but are not limited to the amphiphilic polyanhydride nanoparticles [382], Poly(methyl vinyl ether-co-maleic anhydride) nanoparticles [383], amphiphilic γ-glutamic acid (γ-PGA) nanoparticles [384], cationic lipid nanoparticles [385], ammonio methacrylate copolymer nanoparticles [386], stable nucleic acid lipid particles prepared with cationic lipid-like structures (lipodoids) [387], DiC14-amidine liposomes [388,389], graphene-based nanomaterials [390], and titanium dioxide nanoparticles [391,392]. Alternatively, conventional adjuvants with appropriate physicochemical properties can be incorporated as a structural components of their respective nanoparticulate carriers, thus strengthening the particles’ inherent immunostimulatory properties [374]. Hence, the nanoparticle-mediated combined activation of the TLR and NLRP3 can be exploited to obtain a desirable level of vaccination efficacy [393]. A summary of the nanoparticle-mediated activation of the pro-inflammatory pathways is shown in Figure 6.

A further platform of nanoparticles’ contribution to the field of immunotherapy involves the design of biomimetic nanoparticulate artificial APCs (aAPCs). These particles are tagged with moieties that enable the activation of both the first and the second activating signal on the T cells [394]. Briefly, the first signal is activated through the interaction of the antigen decorated major histocompatibility (MHC) moiety on the nanoparticle surface with the T cell receptor (TCR) expressed by T lymphocytes. The second signal, on the other hand, is simulated through conjugation of the nanoparticle surface with a binder for the T cells’ CD28. Combined, these two signals are able to result in a direct activation of the adaptive immune response [394]. Adjunct to the surface engineered T cell activating moieties, adjustment of the size of these systems has proven necessary for the effective activation of the adaptive immune pathways. For instance, medium sized particles of about 300 nm have been shown to trigger a more decent immune response compared to their small 50 nm counterparts [395]. On the other hand, the possibility of infusion-based administration significantly favors the use of nanosized aAPCs over the similar microscaled systems [396]. Yet considering the non-spherical shape of the natural APCs, adjustment of the nanoparticle shape is also essential to enable an efficient interaction of the surface moieties with the target molecules on the T cell surface [397]. In fact, nanoellipsoidal aAPCs have been shown to exert a superior T cell stimulatory effect compared to their spherical counterparts [398]. Injectable aAPCs have been hitherto successfully exploited for immunotherapeutic purposes in animal models [398,399].

## 8. Concluding Remarks

The advent and development of nanotechnology has made a significant contribution to the development of various aspects of injectable parenteral delivery. While the possibility of active and passive targeting might be considered the major niche of nanotechnology within this arena, nanoparticles have indeed substantially assisted further facets of the art. Beginning with the development stage, nanotechnology has enabled the formulation of numerous low water-soluble APIs as aqueous-based injectables, whose administration as such would be otherwise challenging, if not totally impossible. The nanoparticle platform can be also exploited for the development of locally or systemically injectable long-acting formulations, which will for sure improve the patient compliance. From the biointeraction and biodistribution perspectives, nanoparticles can be modified to have substantial or minimal encounter with the immune system depending on the desired objective. A significant interaction will be of great benefit for immunotherapeutic and vaccination purposes, whereas a minimum exposure is desired for long-acting or targeted formulations. Eventually, passive and active targeting approaches can facilitate the accumulation of the cargo within the desired destination, increasing thereby the ratio of the off-target to on-target drug and improving the patient’s benefit. This can be of course significantly favored by the possibility of a triggered drug release through the design of stimuli-responsive nanocarriers.

The question that presents itself at this stage is to what extent a translation of such nanoformulations from the laboratory to the market would be possible. Notwithstanding the considerable number of the hitherto marketed nanopharmaceuticals (see Table 1), the ratio of the products available in the market to those developed on a laboratory scale remains significantly small. Compared to the APIs formulated as conventional dosage forms, the journey of nanopharmaceuticals from the bench top to the clinic, and subsequently to the market is fraught with complications on various levels. On each level, additional complications are caused by the lack of detailed regulatory guidelines.

The first level involves the material characterization, scale-up and production. Many of the nanoformulations reported in the literature have been prepared using non-FDA approved materials, and through processes that sometimes involve harsh chemicals and relatively unsafe organic solvents [400]. While the development of such systems is an indispensible part of exploring potential therapeutic strategies and expanding the borders of science, the clinical incompatibility of the used materials already restricts the number of the formulations that can be translated into the clinical evaluations. Despite the availability of guidelines regarding the materials for nanoparticle formulation, guidelines specifying the grade and quality of the starting materials is still lacking and should be provided by the regulatory bodies [401].

From the manufacturing perspective, development of nanopharmaceuticals often requires sophisticated processes involving size reduction (e.g., high pressure homogenization, high energy milling, sonication, extrusion, etc.), purification (e.g., organic solvent removal, centrifugation, filtration, etc.), stabilization (e.g., lyophilization, spray-drying, etc.), sterilization, and so forth [402]. Conventionally, many pharmaceutical companies are not equipped with such facilities even for the production of the simplest nanocarrier systems, let alone for the manufacturing of the more complex multifunctionalized formulations. It is hence not surprising that the majority of the hitherto marketed nanopharmaceuticals are simple, non-functionalized delivery units. Additionally, at a larger scale, development of a robust manufacturing with minimal batch-to-batch variation remains challenging, for small changes in the manufacturing can account for substantial impact on the products’ critical attributes [403].

The second level includes the limitations associated with the availability of standardized characterization protocols, particularly under the GMP conditions. In fact, nanoparticle characterization techniques and protocols in terms of particle size and surface charge measurements, determination of drug loading, localization and release, and assessment of in vitro and in vivo cytotoxicity require fundamental validation and standardization, a detailed discussion of which has been presented elsewhere [403,404,405].

Last but not least, it is essential to heed that, in many cases, nanotechnology comprises a disease-driven approach for drug delivery and targeting. Accordingly, a lack of in-depth knowledge regarding the disease heterogeneity in patients can lead to lower clinical efficiency of many nanoformulations compared to their preclinical performance [400]. This, of course, has lower relevance should the nanocarrier formulation be used for non-diseased driven purposes such as solubility enhancement or the development of long-acting formulations.

Notwithstanding the above-debated limitations, nanotechnology has indeed rapidly grown within the past few decades. Within this context, the extensively debated contributions of nanotechnology to the field of injectable parenterals have led to a significant increase in the number of the marketed and pipeline injectable nanoproducts. With the growth of our understanding about the disease pathophysiology, development of personalized medicine, advancements in dosage form manufacturing, standardization of the characterization techniques and protocols, and development of detailed regulatory guidelines for nanomedicines, the future decades will hopefully witness the introduction of further contributions of nanotechnology to the platform of injectable formulations.

## Figures and Tables

**Table 1:** Injectable nanomedicine in the market. Adapted with modification from [8,9,10].

Product	Nanocarrier	API	Indication	Function of the Carrier	Approval	Route of Injection
Abelcet^®^ Amphocil^®^ (Markted name outside USA)	Ribbon-like structures of a bilayered membrane and amphotericin B	Amphotericin B	Systemic fungal infection	MPS targeting	FDA 1995–1996	IV
Abraxane^®^	Albumin-paclitaxel conjugates	Paclitaxel	Metastatic breast cancer, non-small-cell lung cancer	Passive tumor targeting	FDA 2005	IV
Adagen^®^	Monomethoxypolyethylene glycol (PEG) covalently attached to the adenosine deaminase	Adenosine deaminase derived from bovine intestine	Enzyme replacement therapy for the treatment of severe combined immunodeficiency disease associated with adenosine deaminase deficiency	Increase of circulation time and reduction of immunogenicity	FDA 1990	IM
Adynovate^®^	PEG-drug conjugate	Recombinant antihemophilic factor	Hemophilia A	Increase of the drug half life and stability	FDA 2016	IV
AmBisome^®^	Liposome	Amphotericin B	Systemic fungal infections, cryptococcal meningitis and visceral leishmaniasis	MPS targeting	FDA 1997	IV
Amphotec^®^	Colloidal dispersion of disc-like particles of amphotericin B and cholesteryl sulfate	Amphotericin B	Invasive aspergillosis in patients with kidney problems or unresponsive to conventional therapy	MPS targeting	FDA 1996	IV
Cimzia^®^	PEGylated antibody	Fab’ fragment of a humanized anti-TNF-alpha antibody	Rheumatoid arthritis, active psoriatic arthritis, active ankylosing spondylitis, moderate-to-severe plaque psoriasis, Crohn’s disease	Increase of circulation time and reduction of immunogenicity	FDA 2008	IV
Copaxone^®^	Polypeptide (average MW 6.4 kDa) composed of four amino acids (glatiramer)	Glatiramer acetate	Relapsing forms of multiple sclerosis	No mechanism attributable to nanosize	FDA 1996	SC
DaunoXome^®^	Liposome	Daunorubicin citrate	AIDS-related Kopsi’s sarcoma	Passive tumor targeting	FDA 1996	IV
DepoCyt^®^	Liposome	Cytarabine	Lymphomatous malignant meningitis	Sustained drug release	FDA 1999	Intraventricular/intrathecal
DepoDur^®^	Liposome	Morphine sulfate	Pain relief	Sustained drug release	FDA 2004	Epidural
Dexferrum^®^	Iron-dextran conjugate	Iron	Iron deficiency in patients with chronic kidney disease	MPS targeting, increase of dosage	FDA 1996	IV
Diprivan^®^	Nanoemulsion	Propofol	Induction and maintenance of anesthesia	Solubility enhancement	FDA 1989	IV
Eligard^®^	Polymeric nanoparticles	Leuprolide acetate	Advanced prostate cancer	Sustained drug release	FDA 2002	SC
Exparel^®^	Liposome	Bupivacaine	Postsurgical analgesia	MPS targeting	FDA 2011	IV
Feridex^®^	Dextran coated supramagenetic oxide nanoparticles	Diagnostic system	Liver and spleen lesion MRI	MPS targeting	FDA 1996	IV
Feraheme™ (Ferumoxytol)	Dextran coated supramagenetic oxide nanoparticles	Iron	Treatment of iron deficient adults with chronic kidney disease	MPS targeting	FDA 2009	IV
Ferrlecit^®^	Sodium ferric gluconate complex in sucrose injection	Iron	Treatment of iron deficient adults with chronic kidney disease	MPS targeting, increase of dosage	FDA 1999	IV
Fungizone^®^	Micellar dispesion (following reconstitution)	Amphotericin B	Systemic fungal infections	Solubility enhancement	FDA 1966	IV
Gendicine^®^	Virosome	p53 gene	Head and neck squamous cell carcinoma	Intracellular and nucleus targeting	People’s Republicof China 2003	Intratumoral injection/Intravascular infusion
Genexol^®^	Micellar dispersion	Paclitaxel	Metastatic breast cancer, pancreatic cancer	Passive tumor targeting	South Korea 2001	IV
Infed^®^	Iron-dextran complex	Iron	Treatment of iron deficient adults with chronic kidney disease	MPS targeting, increase of dosage	FDA 2009	IV/IM
Inflexal^®^ V	Liposome	Influenza virus antigens	Influenza prophylaxis	Intracellular targeting to the cells of the immunity	Switzerland 1997	IV
Invega Sustenna^®^	Nanocrystal	Paliperidone palmitate	Schizophrenia, schizoaffective disorder	Sustained drug release, solubility enhancement	FDA 2009	IM
Kadcyla^®^	Monoclonal antibody-drug conjugate	DM1	Metastatic breast cancer	Passive and active tumor targeting (antibody against human epidermal growth factor receptor-2), redox responsiveness	FDA 2013	IV
Krystexxa^®^	PEG-aptamer conjugate	Pegloticase	Chronic gout	Increase of circulation time and stability, active targeting	FDA 2010	IV
Macugan^®^	Conjugate of PEG and anti vascular epidermal growth factor aptamer	Pegaptinib	Neovascular age related macular degredation	Increase of circulation time and stability, active targeting	FDA 2004	Intravitreal
Marqibo^®^	Liposome	Vincristine sulfate	Acute lymphoid leukemia, relapsed or progressed Philadelphia chromosome-negative,	Passive tumor targeting	FDA 2012	IV
Mepact™	Liposomes	Mifamurtide	Non-metastasizing resectable osteosarcoma	MPS targeting	Europe 2009	IV
Mircera^®^	methoxy polyethylene glycol-epoetin beta conjugate	Epoetin beta	Treatment of iron deficient adults with chronic kidney disease	Increase of stability	FDA 2007	IV
MM-398	Liposomes	Irinotecan	Treatment of iron deficient adults with chronic kidney disease	Passive tumor targeting	FDA 2015	IV
Myocet^®^	Liposomes	Doxorubicin	Metastatic breast cancer	MPS targeting and formation of MPS depots for slow drug release	Europe 2000	IV
NanoTherm^®^	Aminosilane-coated superparamagnetic iron oxide nanoparticles	Supramagnetic iron oxide nanoparticles	Glioblastoma, prostate and pancreatic cancer	Local tumor ablation under exposure to alternating magnetic field	Europe 2013	Intratumoral
Neulasta^®^	PEG-filgrastim conjugate	Filgrastim (granulocyte colony-stimulating factor)	Febrile neutropenia, In patients with nonmyeloid malignancies; prophylaxis	Increase of protein stability	FDA 2002	SC
Oncaspar^®^	PEG-L-asparaginase conjugate	L-asparaginase	Acute lymphoblastic leukemia	Increase of protein stability and circulation time	FDA 1994	IV/IM
Onivyde^®^	Liposome	Irinotecan	Pancreatic cancer	Passive tumor targeting	FDA 2015	IV
Ontak^®^	Protein (denileukin)-drug conjugate	Recombinant fusion protein of fragment A of diphtheria toxin (diftitox)	Primary cutaneous T-cell lymphoma, CD25-positive, persistent or recurrent disease	Intracellular targeting and lysosomal escape	FDA 1994/2006	IV
Opaxio^®^	Drug conjugated polymeric nanoparticles	Paclitaxel	Glioblastoma	Passive tumor targeting	FDA 2012	IV
Pegasys^®^	PEG-interferon alpha-2a conjugate	Interferon alpha-2a	Hepatitis B and C	Increase of circulation time and stability	FDA 2002	IV
PegIntron^®^	PEG-interferon alphaa-2b conjugate	Interferon alpha-2b	Hepatitis C	Increase of circulation time and stability	FDA 2001	IV
Plegridy^®^	PEG-interferon beta-1a conjugate	Interferon beta-1a	Multiple sclerosis	Increase of circulation time and stability	FDA 2014	IV
Rebinyn^®^	GlycoPEG-recombinant coagulation factor IX conjugate	Recombinant coagulation factor IX	Hemophilia B	Increase of drug half life and Cmax	FDA 2017	IV
Rexin-G^®^	Virosome	Gene for dominant-negative mutant form of human cycline G1	Solid tumors	Intracellular and nucleus targeting	Philippines 2007	IV
Ryanodex^®^	Nanocrystal	Dantrolene sodium	Acromegaly	Increase of the administration rate and dosis	FDA 2003	IV
Somavert^®^	PEG-human growth hormone receptor antagonist conjugate	Pegvisomant	Acute lymphoblastic leukemia	Increase of protein stability and circulation time	FDA 1994	IV/IM
Venofer t^®^	Iron-sucrose complex	Iron	Treatment of iron deficient adults with chronic kidney disease	Increase of administrable dose	FDA 2000	IV
Visudyne^®^	Liposome	Verteporfin	Osteoarthritis knee pain	Solubility enhancement	FDA 2000	IV
Vyxeos^®^	Liposome	Daunorubicin and cytarabine	Acute myeloid leukemia	Passive tumor targeting	FDA 2017	IV
Zilretta^®^	Polymeric microparticles with nanosized pores	Triamcinolone acetonide	Primary cutaneous T-cell lymphoma, CD25-positive, persistent or recurrent disease	Sustained drug release	FDA 2017	Intra-arterial
Zinostatin stimalamer^®^	Protein/copolymer of styrene-maleic acid-NCS conjugate	Antitumor protein NCS	Primary unresectable hepatocellular carcinoma	Passive tumor targeting	Japan 1994	IV

**Table 2:** Nanoparticle-based injectable formulations currently in clinical trials.

Intervention	Nanosystem	Associated API	Function of the Nanosystem	Condition	Route of Injection	Stage of Evaluation	Status	Identifier
ABI-007 + Gemcitabin	Albumin stabilized NPs	Paclitaxel	Tumor targeting, drug solubility enhancement	- Metastatic breast cancer	IV	Phase II	Completed	NCT00110084
ABI-007	Albumin stabilized NPs	Paclitaxel	Tumor targeting, drug solubility enhancement	- Non-small cell lung cancer	IV	Phase I and II	Completed	NCT00073723
BIND-014	PEG-coated, PSMA targeted poly lactic NPs	Docetaxel	Passive and active tumor targeting, drug solubility enhancment	- Metastatic cancer - Cancer - Solid tumors	IV	Phase I	Completed	NCT01300533
BIND-014	PEG-coated, PSMA targeted poly lactic NPs	Docetaxel	Passive and active tumor targeting, drug solubility enhancment	- KRAS positive patients with non-small cell lung cancer - Squamous cell non-small cell lung cancer	IV	Phase II	Completed	NCT02283320
BIND-014	PEG-coated, PSMA targeted poly lactic NPs	Docetaxel	Passive and active tumor targeting, drug solubility enhancment	- Urothelial carcinoma - Cholangiocarcinoma - Cervical cancer - Squamous cell carcinoma of head and neck	IV	Phase II	Terminated	NCT02479178
BIND-014	PEG-coated, PSMA targeted poly lactic NPs	Docetaxel	Passive and active tumor targeting, drug solubility enhancment	- Non-small cell lung cancer	IV	Phase II	Completed	NCT01792479
BIND-014	PEG-coated, PSMA targeted poly lactic NPs	Docetaxel	Passive and active tumor targeting, drug solubility enhancement	- Metastatic Castration-Resistant Prostate Cancer	IV	Phase II	Completed	NCT01812746
C19-A3 GNP	Gold NPs	C19-A3 peptide	Enhanced APC uptake	Type 1 diabetes	Intradermal	Phase I	Active/not recruiting	NCT02837094
Ceramide nanoliposome	Nanoliposome	Ceramide (non-conventional)	Passive tumor targeting	- Cancer - Carcinoma - Solid tumors	IV	Phase I	Recruiting	NCT02834611
CRLX101	Drug-linear cyclodextrin–PEG copolymer conjugate	Camptothecin	Tumor targeting, drug solubility enhancement	- Extensive stage small cell lung cancer - Recurrent small cell lung cancer	IV	Phase II	Terminated	NCT01803269
CRLX101 + Olaparib	Drug-linear cyclodextrin–PEG copolymer conjugate	Camptothecin	Tumor targeting, drug solubility enhancement	- Solid tumors - Small cell lung carcinoma - Non-small-cell lung carcinoma - Lung neoplasms - Small cell lung cancer - Lung cancer	IV	Phase I and II	Recruiting	NCT02769962
ND-L02-s0201 (BMS-986263)	Vitamin A-moieties conjugated lipid NP	HSP47siRNA	Passive and active hepatic targeting, intracellular delivery, increase of the cargo’s stability	- Moderate to extensive hepatic fibrosis	IV	Phase I	Completed	NCT02227459
Nab-paclitaxel	Albumin stabilized NPs	Paclitaxel	Passive tumor targeting, drug solubility enhancement	- Intraocular melanoma	IV	Phase II	Completed	NCT00738361
Nab-paclitaxel	Albumin stabilized NPs	Paclitaxel	Tumor targeting, drug solubility enhancement	- Metastatic Breast Cancer	IV	Phase II	Terminated	NCT01416558
Nab-paclitaxel	Albumin stabilized NPs	Paclitaxel	Ttumor targeting, drug solubility enhancement	- Vascular Disease - Peripheral	IV	Phase II	Terminated	NCT00518284
Nab-paclitaxel + Durvalumab + Carboplatin + Cisplatin	Albumin stabilized NPs	Paclitaxel	Tumor targeting, drug solubility enhancment	- Carcinoma - Squamous cell - Oral cancer - Oropharynx cancer - Larynx cancer - Lip cancer - Esophageal cancer	IV	Phase II	Recruiting	NCT03174275
Nab-paclitaxel + Pembrolizumab + Epirubicin + Cyclophosphamide	Albumin stabilized NPs	Paclitaxel	Tumor targeting, drug solubility enhancement	- Malignant neoplasm of breast	IV	Phase II	Active/not recruiting	NCT03289819
Nab-paclitaxel + Pembrolizumab + Carboplatin	Albumin stabilized NPs	Paclitaxel	Tumor targeting, drug solubility enhancement	- Non-small cell lung cancer	IV	Phase III	Active/not recruiting	NCT02775435
Nab-paclitaxel + Sargramostim	Albumin stabilized NPs	Paclitaxel	Tumor targeting, drug solubility enhancement	- Brenner tumor - Fallopian tube cancer - Ovarian clear cell cystadenocarcinoma - Ovarian endometrioid adenocarcinoma - Ovarian mixed epithelial carcinoma - Ovarian mucinous cystadenocarcinoma - Ovarian serous cystadenocarcinoma - Ovarian undifferentiated adenocarcinoma - Peritoneal cavity cancer - Recurrent ovarian epithelial cancer - Stage III ovarian epithelial cancer - Stage IV ovarian epithelial cancer	IV	Phase II	Completed	NCT00466960
NanoFlu	Recombinant hemagglutinin NPs	Recombinant hemagglutinin (antigen), Matrix-M™ adjuvant	Passive immune cell targeting, increase of immune stimulation	- Influenza prophylaxis	IM	Phase I and II	Completed	NCT03293498
Nanoliposomal irinotecan	Nanoliposomes	Irinotecan	Tumor targeting	-High Grade Glioma	IV	Phase I	Enrolling by invitation	NCT02022644
RXDX-107	Albumin NPs	Bendamustine derivative (dodecanol alkyl ester)	Tumor targeting, Macropinocytosis mediated intracellular delivery	- Solid tumors	IV	Phase I	Terminated	NCT02548390
STP705	Polypeptide nanoparticle	Anti-fibrosis and anti-inflammatory siRNA	Enhanced targeted intracellular delivery	- Hypertrophic scar	Intradermal	Phase I and II	Recruiting	NCT02956317

Abbreviations: KRAS: Kirsten rat sarcoma viral oncogene homolog, Nab: nano-albumin, NP: nanoparticle, PSMA: prostate cancer membrane antigen, siRNA: small interfering RNA.

**Table 3:** Examples recently developed pH-responsive nanocarriers with potentials for parenteral drug delivery.

Type of the Nanocarrier	pH Responsive Moiety	Incorporated Cargo	Application	Reference
Layer-by-layer assembled nanoparticles	Neutravidin-iminobiotin bond	Quantum Dots	Stealth coating shedding, cancer therapy	[181]
Lipid core nanoparticles	polyethylene glycol-*b*-polyaspartic acid	Docetaxel	Stealth coating shedding, cancer therapy	[182]
Polymeric nanospheres	poly- (1,4-phenyleneacetone dimethylene ketal)	Dexamethasone	Intracellular drug release	[184]
Drug-polymer conjugate	Cleavable amide bond	Doxorubicin	Intracellular drug release, cancer therapy	[187]
Drug-polymer conjugate	Hydrazone bond	Doxorubicin	Intracellular drug release, cancer therapy	[188]
Cyclodextrin-derived nanoparticles	Poly(cyclohexane-1, 4-diyl acetone dimethylene ketal)	Paclitaxel	Intracellular drug release, cancer therapy	[189]
Drug-polymer conjugate	Hydrozone bond	Cisplatin	Intracellular drug release, cancer therapy	[190]
Mesoporpous silica nanoparticle	Hydrozone bond	Doxorubicin	Intracellular drug release, cancer therapy	[191]
Polymeric micelles	Poly(β-amino ester)	Doxorubicin	Intracellular drug release, cancer therapy	[192]
Nanogels	Amino groups	Oridonin	Intracellular drug release, Drug release in tumor extracellular environment, cancer therapy	[193]
Polymeric micelles	N-Boc-histidine	Doxorubicin	Drug release in tumor extracellular environment, cancer therapy	[179]
Polymeric micelles	Poly(β-amino ester)	Doxorubicin	Drug release in tumor extracellular environment, cancer therapy	[180]
Polymeric nanoparticles	Chitosan	Camptothecin	Drug release in tumor extracellular environment, cancer therapy	[194]
polymeric micelles	poly(beta-amino ester)	Doxorubicin	Drug release in tumor extracellular environment, cancer therapy	[195]
Flower-like polymeric micelle	poly(DEAP-Lys)	Doxorubicin	Drug release in tumor extracellular environment, cancer therapy	[196]
Micelle-like nanoparticles	Poly(*N*- methacryloyl-l-valine) and poly(*N*-methacryloyl-L-phenylala- nine)	-	-	[197]

## References

[1] Gulati N., Gupta H. (2011). Parenteral drug delivery: A review. Recent Pat. Drug Deliv. Formul..

[2] Vega J.A., Ochoa P.S., Holder P. (2015). Introduction to parenteral preparations. Concepts in Steril Preparations and Aseptic Techniques.

[3] Packhaeuser C.B., Schneider J., Oster C.G., Kissel T. (2004). In situ forming parenteral drug delivery systems: An overview. Eur. J. Pharm. Biopharm..

[4] Joshi M.D., Müller R.H. (2009). Lipid nanoparticles for parenteral delivery of actives. Eur. J. Pharm. Biopharm..

[5] Patravale V., Prajakta D., Jain R. (2012). Nanoparticles as drug carriers. Nanoparticulate Drug Delivery: Perspectives on the Transition from Laboratory to Market.

[6] Bari H. (2010). A prolonged release parenteral drug delivery system—An overview. Int. J. Pharm. Sci. Rev. Res..

[7] Ogawara K., Yoshizawa Y., Un K., Araki T., Kimura T., Higaki K. (2013). Nanoparticle-based passive drug targeting to tumors: Considerations and implications for optimization. Biol. Pharm. Bull..

[8] Weissig V., Pettinger T.K., Murdock N. (2014). Nanopharmaceuticals (part 1): Products on the market. Int. J. Nanomed..

[9] Ragelle H., Danhier F., Préat V., Langer R., Anderson D.G. (2017). Nanoparticle-based drug delivery systems: A commercial and regulatory outlook as the field matures. Expert Opin. Drug Deliv..

[10] Ventola C.L. (2017). Progress in nanomedicine: Approved and investigational nanodrugs. Pharm. Ther..

[11] Kalepu S., Nekkanti V. (2015). Insoluble drug delivery strategies: Review of recent advances and business prospects. Acta Pharm. Sin. B.

[12] Narvekar M., Xue H.Y., Eoh J.Y., Wong H.L. (2014). Nanocarrier for poorly water-soluble anticancer drugs—Barriers of translation and solutions. AAPS Pharmscitech.

[13] Tuomela A., Hirvonen J., Peltonen L. (2016). Stabilizing agents for drug nanocrystals: Effect on bioavailability. Pharmaceutics.

[14] Kipp J.E. (2004). The role of solid nanoparticle technology in the parenteral delivery of poorly water-soluble drugs. Int. J. Pharm..

[15] Mohammed A.R., Weston N., Coombes A.G.A., Fitzgerald M., Perrie Y. (2004). Liposome formulation of poorly water soluble drugs: Optimisation of drug loading and ESEM analysis of stability. Int. J. Pharm..

[16] Nagarwal R.C., Kumar R., Dhanawat M., Das N., Pandit J.K. (2011). Nanocrystal technology in the delivery of poorly soluble drugs: An overview. Curr. Drug Deliv..

[17] Hörmann K., Zimmer A. (2016). Drug delivery and drug targeting with parenteral lipid nanoemulsions—A review. J. Control. Release Off. J. Control. Release Soc..

[18] Hoskins C., Cheng W.P. (2012). Implementing nanotechnology and novel drug delivery systems to improve dissolution and solubilization. Am. Pharm. Rev..

[19] Trivedi R., Kompella U.B. (2010). Nanomicellar formulations for sustained drug delivery: Strategies and underlying principles. Nanomedicine.

[20] Greco F., Vicent M.J. (2008). Polymer-drug conjugates: Current status and future trends. Front. Biosci. J. Virtual Libr..

[21] Larson N., Ghandehari H. (2012). Polymeric conjugates for drug delivery. Chem. Mater. Publ. Am. Chem. Soc..

[22] Li C., Wallace S. (2008). Polymer-drug conjugates: Recent development in clinical oncology. Adv. Drug Deliv. Rev..

[23] Rangel-Yagui C.O., Junior A.P., Tavares L. (2005). Micellar solubilization of drugs. J. Pharm. Pharm. Sci..

[24] Gupta U., Agashe H.B., Jain N.K. (2007). Polypropylene imine dendrimer mediated solubility enhancement: Effect of pH and functional groups of hydrophobes. J. Pharm. Pharm. Sci..

[25] Jain N.K., Gupta U. (2008). Application of dendrimer-drug complexation in the enhancement of drug solubility and bioavailability. Expert Opin. Drug Metab. Toxicol..

[26] Svenson S., Chauhan A.S. (2008). Dendrimers for enhanced drug solubilization. Nanomedicine.

[27] Siddalingappa B., Nekkanti V., Betageri G. (2013). Insoluble drug delivery technologies: Review of health benefits and business potentials. OA Drug Des. Deliv..

[28] Gidwani B., Vyas A. (2015). A comprehensive review on cyclodextrin-based carriers for delivery of chemotherapeutic cytotoxic anticancer drugs. BioMed Res. Int..

[29] Lakkakula J.R., Maçedo Krause R.W. (2014). A vision for cyclodextrin nanoparticles in drug delivery systems and pharmaceutical applications. Nanomedicine.

[30] Hierrezuelo J., Pelaez L., Benavente J., Lopez-Romero J.M., Rico R., Armengol R. (2014). Lipid and cyclodextrin nanocarriers loading bioactive agents: Stabilization on polymeric supports. Bioengineered Nanomaterials.

[31] Nasir A., Harikumar S., Amanpreet K. (2012). Cyclodextrins: An excipient tool in drug delivery. Int. Res. J. Pharm..

[32] Tejashri G., Amrita B., Darshana J. (2013). Cyclodextrin based nanosponges for pharmaceutical use: A review. Acta Pharm. Zagreb Croat..

[33] Olteanu A.A., Arama C.C., Mihaescu C., Moniciu C.-M. (2014). Effect of β-cyclodextrins based nanosponges on the solubility of lipophilic pharmacological active substances (repaglinide). J. Incl. Phenom. Macrocycl. Chem..

[34] Hu Q.-D., Tang G.-P., Chu P.K. (2014). Cyclodextrin-based host-guest supramolecular nanoparticles for delivery: From design to applications. Acc. Chem. Res..

[35] Zerkoune L., Angelova A., Lesieur S. (2014). Nano-assemblies of modified cyclodextrins and their complexes with guest molecules: Incorporation in nanostructured membranes and amphiphile nanoarchitectonics design. Nanomaterials.

[36] Oommen E., Dinesh Shenoy B., Udupa N., Kamath R., Uma Devi P. (1999). Antitumour efficiency of cyclodextrin-complexed and niosome-encapsulated plumbagin in mice bearing melanoma. Pahrmacy Pharmacol. Commun..

[37] Yallapu M.M., Ebeling M.C., Khan S., Sundram V., Chauhan N., Gupta B.K., Puumala S.E., Jaggi M., Chauhan S.C. (2013). Novel curcumin-loaded magnetic nanoparticles for pancreatic cancer treatment. Mol. Cancer Ther..

[38] Namgung R., Mi Lee Y., Kim J., Jang Y., Lee B.-H., Kim I.-S., Sokkar P., Rhee Y.M., Hoffman A.S., Kim W.J. (2014). Poly-cyclodextrin and poly-paclitaxel nano-assembly for anticancer therapy. Nat. Commun..

[39] Jiang Y., Liang M., Svejkar D., Hart-Smith G., Lu H., Scarano W., Stenzel M.H. (2014). Albumin-micelles via a one-pot technology platform for the delivery of drugs. Chem. Commun..

[40] Karimi M., Bahrami S., Baghaee Ravari S., Sahandi P., Mirshekari H., Bozorgomid M., Shahreza S., Sori M., Hamblin M.R. (2016). Albumin nanostructures as advanced drug delivery systems. Expert Opin. Drug Deliv..

[41] Naveen R., Akshata K., Pimple S., Chaudhari P. (2016). A review on albumin as drug carrier in treating different diseases and disorders. Pharm. Sin.

[42] Chen H., Khemtong C., Yang X., Chang X., Gao J. (2011). Nanonization strategies for poorly water-soluble drugs. Drug Discov. Today.

[43] Khadka P., Ro J., Kim H., Kim I., Kim J.T., Kim H., Cho J.M., Yun G., Lee J. (2014). Pharmaceutical particle technologies: An approach to improve drug solubility, dissolution and bioavailability. Asian J. Pharm..

[44] Möschwitzer J.P. (2013). Drug nanocrystals in the commercial pharmaceutical development process. Int. J. Pharm..

[45] Sun B., Yoe Y. (2012). Nanocrystals for the parenteral delivery of poorly water-soluble drugs. Curr. Opin. Solid State Mater. Sci..

[46] Katteboinaa S., Chandrasekhar P., Balaji S. (2009). Drug nanocrystals: A novel formulation approach for poorly soluble drugs. Int. J. PharmTech Res..

[47] Wang Y., Ma Y., Ma Y., Du Y., Liu Z., Zhang D., Zhang Q. (2012). Formulation and pharmacokinetics evaluation of puerarin nanocrystals for intravenous delivery. J. Nanosci. Nanotechnol..

[48] Gu L., Fang R.H., Sailor M.J., Park J.-H. (2012). In Vivo clearance and toxicity of monodisperse iron oxide nanocrystals. ACS Nano.

[49] Mouton J., van Peer A., de Beule K., Van Vliet A., Donnelly J., Soons P. (2006). Pharmacokinetics of itraconazole and OH-itraconazole as a nanocrystal formulation in healthy volunteers after single and multiple doses|Aspergillus & Aspergillosis Website. Antimicrob. Agents Chemother..

[50] Wei L., Ji Y., Gong W., Kang Z., Meng M., Zheng A. (2015). Preparation, physical characterization and pharmacokinetic study of paclitaxel nanocrystals. Drug Dev. Ind. Pharm..

[51] Lu Y., Wang Z., Li T., McNally H., Park K., Sturek M. (2015). Development and evaluation of transferrin-stabilized paclitaxel nanocrystal formulation. J. Control. Release.

[52] Hollis C.P., Weiss H.L., Leggas M., Evers B.M., Gemeinhart R.A., Li T. (2013). Biodistribution and bioimaging studies of hybrid paclitaxel nanocrystals: Lessons learned of the EPR effect and image-guided drug delivery. J. Control. Release Off. J. Control. Release Soc..

[53] Chen L., Wang Y., Zhang J., Hao L., Guo H., Lou H., Zhang D. (2014). Bexarotene nanocrystal—Oral and parenteral formulation development, characterization and pharmacokinetic evaluation. Eur. J. Pharm. Biopharm..

[54] Chiang P.-C., Ran Y., Chou K.-J., Cui Y., Wong H. (2011). Investigation of utilization of nanosuspension formulation to enhance exposure of 1,3-dicyclohexylurea in rats: Preparation for PK/PD study via subcutaneous route of nanosuspension drug delivery. Nanoscale Res. Lett..

[55] Xiong R., Lu W., Li J., Wang P., Xu R., Chen T. (2008). Preparation and characterization of intravenously injectable nimodipine nanosuspension. Int. J. Pharm..

[56] Junghanns J.-U.A., Müller R.H. (2008). Nanocrystal technology, drug delivery and clinical applications. Int. J. Nanomed..

[57] Yun Y.H., Lee B.K., Park K. (2015). Controlled drug delivery: Historical perspective for the next generation. J. Control. Release.

[58] Schwendeman S.P., Shah R.B., Bailey B.A., Schwendeman A.S. (2014). Injectable controlled release depots for large molecules. J. Control. Release Off. J. Control. Release Soc..

[59] Karode N.P., Prajapati V.D., Solanki H.K., Jani G.K. (2015). Sustained release injectable formulations: Its rationale, recent progress and advancement. World J. Pharm. Pharm. Sci..

[60] Yang W.-W., Pierstorff E. (2012). Reservoir-based polymer drug delivery systems. J. Lab. Autom..

[61] Kalyani M., Surendra P., Sirisha V. (2013). Parenteral controlled drug delivery system. Int. J. Res. Pharm. Nano Sci..

[62] Larsen C., Weng Larsen S., Jensen H., Jaghmur A., Ostergaard J. (2009). Role of in vitro release models in formulation development and quality control of parenteral depots. Expert Opin. Drug Deliv..

[63] Morais J.M., Burgess D.J. (2012). Micro- and nanoemulsions (controlled release parenteral drug delivery systems). Long Acting Injectables and Implants.

[64] McLennan D.N., Porter C.J., Charman S.A. (2005). Subcotaneous drug delivery and the role of the lymphatics. Drug Discov. Today.

[65] Wei X.-L., Han Y.-R., Quan L.-H., Liu C.-Y., Liao Y.-H. (2013). Oily nanosuspension for long-acting intramuscular delivery of curcumin didecanoate prodrug: Preparation, characterization and In Vivo evaluation. Eur. J. Pharm. Sci. Off. J. Eur. Fed. Pharm. Sci..

[66] Larsen S.W., Østergaard J., Friberg-Johansen H., Jessen M.N.B., Larsen C. (2006). In Vitro assessment of drug release rates from oil depot formulations intended for intra-articular administration. Eur. J. Pharm. Sci. Off. J. Eur. Fed. Pharm. Sci..

[67] Sartorius G., Fennell C., Spasevska S., Turner L., Conway A.J., Handelsman D.J. (2010). Factors influencing time course of pain after depot oil intramuscular injection of testosterone undecanoate. Asian J. Androl..

[68] Rhee Y.-S., Park C.-W., DeLuca P., Mansour H.M. (2010). Sustained-release injectable drug delivery. Pharm. Technol..

[69] Jogala S., Rachamalla S.S., Aukunuru J. (2015). Development of subcotaneous sustained release nanoparticles encapsulating low molecular weight heparin. J. Adv. Pharm. Technol. Res..

[70] Hassan A.H., Hosny K.M., Alhadlaq A., Alyamani A., Naguib G. (2015). Depot injectable biodegradable nanoparticles loaded with recombinant human bone morphogenetic protein-2: Preparation, characterization, and In Vivo evaluation. Drug Des. Devel. Ther..

[71] Xu X., Wang Y., Chen R., Feng C., Yao F., Tong S., Wang L., Yamashita F., Yu J. (2011). Formulation and pharmacokinetic evaluation of tetracycline-loaded solid lipid nanoparticles for subcotaneous injection in mice. Chem. Pharm. Bull..

[72] Suitthimeathegorn O., Turton J.A., Mizuuchi H., Florence A.T. (2007). Intramuscular absorption and biodistribution of dexamethasone from non-aqueous emulsions in the rat. Int. J. Pharm..

[73] Allen T.M., Hansen C.B., Peliowski A. (1993). Subcutaneous administration of sterically stabilized (stealth) liposomes is an effective sustained release system for 1-β-d-arabinofuranosylcytosine. Drug Deliv..

[74] Corvo M.L., Boerman O.C., Oyen W.J., Jorge J.C., Cruz M.E., Crommelin D.J., Storm G. (2000). Subcutaneous administration of superoxide dismutase entrapped in long circulating liposomes: In Vivo fate and therapeutic activity in an inflammation model. Pharm. Res..

[75] Freeling J.P., Koehn J., Shu C., Sun J., Ho R.J. (2014). Long-acting three-drug combination anti-HIV nanoparticles enhance drug exposure in primate plasma and cells within lymph nodes and blood. AIDS.

[76] Kamaly N., Xiao Z., Valencia P.M., Radovic-Moreno A.F., Farokhzad O.C. (2012). Targeted polymeric therapeutic nanoparticles: Design, development and clinical translation. Chem. Soc. Rev..

[77] Liu R., Priestley R.D. (2016). Rational design and fabrication of core–shell nanoparticles through a one-step/pot strategy. J. Mater. Chem. A.

[78] Mandal B., Bhattacharjee H., Mittal N., Sah H., Balabathula P., Thoma L.A., Wood G.C. (2013). Core-shell-type lipid-polymer hybrid nanoparticles as a drug delivery platform. Nanomed. Nanotechnol. Biol. Med..

[79] Park J., Wrezesinski S.H., Stern E., Look M., Criscione J., Ragheb R., Jay S.M., Demento S.L., Agawu A., Limon P.L. (2012). Combination delivery of TGF-b inhibitor and IL-2 by nanoscale liposomal polymeric gels enhances tumor immunotherapy. Nat. Mater..

[80] Chan J.M., Zhang L., Yuet K.P., Liao G., Rhee J.-W., Langer R., Farokhzad O.C. (2009). PLGA-Lecithin-PEG core-shell nanoparticles for controlled drug delivery. Biomaterials.

[81] Haidar Z.S., Hamdy R.C., Tabrizian M. (2008). Protein release kinetics for cor-shell hybrid nanoparticles based on the layer-by-layer assembly of alginate and chitosan on liposomes. Biomaterials.

[82] Mittapelly N., Rachumallu R., Pandey G., Sharma S., Arya A., Bhatta R.S., Mishra P.R. (2016). Investigation of the salt formation between memantine and pamoic acid: Its exploitation in nanocrystalline form as long-acting injection. Eur. J. Pharm. Biopharm..

[83] Hu L., Yang C., Kong D., Gao N., Zhai F. (2016). Development of a long-acting intramuscularly injectable formulation with nanosuspension of andrographolide. J. Drug Deliv. Sci. Technol..

[84] Baert L., van Klooster G., Dries W., Francois M., Wouters A., Basstanie E., Iterbeke K., Stappers F., Stevens P., Schueller L. (2009). Development of a long-acting injectable formulation with nanoparticles of rilpivirine (TMC278) for HIV treatment. Eur. J. Pharm. Biopharm..

[85] Hollis C.P. (2012). Nanocrystals of Chemotherapeutic Agents for Cancer Theranostics: Development and In Vitro and In Vivo Evaluation.

[86] Kempe S., Mäder K. (2012). In Situ forming implants—An attractive formulation principle for parenteral depot formulations. J. Control. Release Off. J. Control. Release Soc..

[87] Hatefi A., Amsden B. (2002). Biodegradable injectable in situ forming drug delivery systems. J. Control. Release Off. J. Control. Release Soc..

[88] Thoniyot P., Tan M.J., Karim A.A., Young D.J., Loh X.J. (2015). Nanoparticle–hydrogel composites: Concept, design, and applications of these promising, multi-functional materials. Adv. Sci..

[89] Shi Y., Li L.C. (2005). Current advances in sustained-release systems for parenteral drug delivery. Expert Opin. Drug Deliv..

[90] Packhaeuser C.B., Kissel T. (2007). On the design of in situ forming biodegradable parenteral depot systems based on insulin loaded dialkylaminoalkyl-amine-poly(vinyl alcohol)-g-poly(lactide-co-glycolide) nanoparticles. J. Control. Release Off. J. Control. Release Soc..

[91] Yu L., Ding J. (2008). Injectable hydrogels as unique biomedical materials. Chem. Soc. Rev..

[92] Amin S., Rajabnezhad S., Kohli K. (2009). Hydrogels as potential drug dleivery systems. Sci. Res. Essay.

[93] Lin C.-C., Metters A.T. (2006). Hydrogels in controlled release formulations: Network design and mathematical modeling. Adv. Drug Deliv. Rev..

[94] Patel A., Cholkar K., Mitra A.K. (2014). Recent developments in protein and peptide parenteral delivery approaches. Ther. Deliv..

[95] Grijalvo S., Mayr J., Eritja R., Diaz Diaz D. (2016). Biodegradable liposome-encapsulated hydrogels for biomedical applications: A marriage of convenience. Biomater. Sci..

[96] Zhao F., Yao D., Ruiwei G., Deng L., Dong A., Zhang J. (2015). Composites of polymer hydrogels and nanoparticulate systems for biomedical and pharmaceutical applications. Nanomaterials.

[97] Mishra G.P. (2006). Development and Evaluation of Novel In Situ Depot-Forming Controlled Release Formulations. Ph.D. Thesis.

[98] Appel E.A., Tibbitt M.W., Webber M.J., Mattix B.A., Veiseh O., Langer R. (2015). Self-assembled hydrogels utilizing polymer–nanoparticle interactions. Nat. Commun..

[99] Satarkar N.S., Biswal D., Hilt J.Z. (2010). Hydrogel nanocomposites: A review of applications as remote controlled biomaterials. Soft Matter.

[100] Liu J., Zhang S.M., Chen P.P., Cheng L., Zhou W., Tang W.X., Chen Z.W., Ke C.M. (2007). Controlled release of insulin from PLGA nanoparticles embedded within PVA hydrogels. J. Mater. Sci. Mater. Med..

[101] Peng Q., Sun X., Gong T., Wu C.-Y., Zhang T., Tan J., Zhang Z.-R. (2013). Injectable and biodegradable thermosensitive hydrogels loaded with PHBHHx nanoparticles for the sustained and controlled release of insulin. Acta Biomater..

[102] Ciobanu B.C., Cadinoiu A.N., Popa M., Desbrieres J., Peptu C.A. (2014). Modulated release from liposomes entapped in chitosan/gelatin hydrogels. Mater. Sci. Eng. C.

[103] Chung Y.-I., Ahn K.-M., Jeon S.-Y., Lee J.-H., Tae G. (2007). Enhanced bone generation with BMP-2 loaded functional nanoparticle-hydrogel complex. J. Control. Release.

[104] Alinaghi A., Rouini M., Johari Daha F., Moghimi H. (2013). Hydrogel-embeded vesicles as a novel approach for prolonged release and delivery of liposomes In Vitro and In Vivo. J. Liposome Res..

[105] Dorraj G., Moghimi H.R. (2015). Preparation of SLN-containing thermoresponsive in-situ Forming gel as a controlled nanoparticle delivery system and investigating its rheological, thermal and erosion behavior. Iran. J. Pharm. Res. Ijpr.

[106] Hamidi M., Azadi A., Rafiei P. (2008). Hydrogel nanoparticles in drug delivery. Adv. Drug Deliv. Rev..

[107] Goncalves C., Pereira P., Gama M. (2010). Self-assembled hydrogel nanoparticles for drug delivery. Materials.

[108] Arnfast L., Madsen C.G., Jorgensen L., Baldursdottir S. (2014). Design and processing of nanogels as delivery systems for peptides and proteins. Ther. Deliv..

[109] Akiyoshi K., Kobayashi S., Shichibe S., Mix D., Baudys M., Kim S.W., Sunamoto J. (1998). Self-assembeled hydrogel nanoparticle of cholesterol-bearing pllulan as a carrier of protein drugs: Complexation and stabilization of insulin. J. Control. Release.

[110] Shimizu T., Kishida T., Hasegawa U., Ueda Y., Imanishi J., Yamagishi H., Akiyoshi K., Otsuji E., Mazda O. (2008). Nanogel DDS enables sustained release of IL-12 for tumor immunotherapy. Biochem. Biophys. Res. Commun..

[111] Kitano S., Kageyama S., Nagata Y., Miyahara Y., Hiasa A., Naota H., Okumura S., Imai H., Shiraishi T., Masuya M. (2006). HER2-specific T-cell immune responses in patients vaccinated with truncated HER2 protein complexed with nanogels of cholesteryl pullulan. Clin. Cancer Res. Off. J. Am. Assoc. Cancer Res..

[112] Kageyama S., Kitano S., Hirayama M., Nagata Y., Imai H., Shiraishi T., Akiyoshi K., Scott A.M., Murphy R., Hoffman E.W. (2008). Humoral immune responses in patients vaccinated with 1-146 HER2 protein complexed with cholesteryl pullulan nanogel. Cancer Sci..

[113] Shimoda A., Yamamoto Y., Sawada S., Akiyoshi K. (2012). Biodegradable nanogel-integrated hydrogels for sustained protein delivery. Macromol. Res..

[114] Hoare T., Young S., Lawlor M.W., Kohane D.S. (2012). Thermoresponsive nanogels for prolonged duration local anesthesia. Acta Biomater..

[115] Blanco M.D., Guerrero S., Benito M., Jaghmur A., Teijon C., Olmo R., Katime I., Teijon J.M. (2011). In Vitro and In Vivo evaluation of a folate-targeted copolymeric submicrohydrogel based on N-isopropylacrylamide as 5-fluorouracil delivery system. Polymers.

[116] Hirlekar R., Jain S., Patel M., Garse H., Kadam V. (2010). Hexosomes: A novel drug delivery system. Curr. Drug Deliv..

[117] Boyd B.J., Whittaker D.V., Khoo S.-M., Davey G. (2006). Hexosomes formed from glycerate surfactants—Formulation as a colloidal carrier for irinotecan. Int. J. Pahrmaceutics.

[118] Omray L. (2013). Liquid crystals as novel vesicular delivery system: A review. Curr. Trends Technol. Sci..

[119] Boyd B.J., Whittaker D.V., Khoo S.-M., Davey G. (2006). Lyotropic liquid crystalline phases formed from glycerate surfactants as sustained release drug delivery systems. Int. J. Pharm..

[120] Phan S., Fong W.-K., Kirby N., Hanley T., Boyd B.J. (2011). Evaluating the link between self-assembled mesophase structure and drug release. Int. J. Pharm..

[121] Couffin-Hoarau A.-C., Motulsky A., Delmas P., Leroux J.-C. (2004). In Situ-forming pharmaceutical organogels based on the self-assembly of L-alanine derivatives. Pharm. Res..

[122] Angelova A., Angelov B., Mutafchieva R., Lesieur S., Couvreur P. (2011). Self-assembled multicompartment liquid crystalline lipid carriers for protein, peptide, and nucleic acid drug delivery. Acc. Chem. Res..

[123] Chen Y., Ma P., Gui S. (2014). Cubic and hexagonal liquid crystals as drug delivery systems. BioMed Res. Int..

[124] Ki M.-H., Lim J.-L., Ko J.-Y., Park S.-H., Kim J.-E., Cho H.-J., Park E.-S., Kim D.-D. (2014). A new injectable liquid crystal system for one monthe delivery of leuprolide. J. Control. Release.

[125] Rizwan S., McBurney W., Young K., Hanley T., Boyd B.J., Rades T., Hook S. (2013). Cubosomes containing adjuvants imiquimod and monophosphoryl lipid A stimulate robust cellular and humoral immune responses. J. Control. Release.

[126] Nasr M., Ghorab M.K., Abdelazem A. (2015). In Vitro and In Vivo evaluation of cubosomes containing 5-fluorouracil for liver targetting. Acta Pharm. Sin. B.

[127] Liu Z., Luo L., Zheng S., Niu Y., Bo R., Huang Y., Xing J., Li Z., Wang D. (2016). Cubosome nanoparticles potentiate immune properties of immunostimulants. Int. J. Nanomed..

[128] Jogala S., Rachamalla S.S., Aukunuru J. (2016). Development of PEG-PLGA based intravenous low molecular wight heparin (LMWH) nanoparticles intended to treat venous thrombosis. Curr. Drug Deliv..

[129] Li Y., Pei Y., Zhang X., Gu Z., Zhou Z., Yuan W., Zhou J., Zhu J., Gao X. (2001). PEGylated PLGA nanoparticles as protein carriers: Synthesis, preparation and biodistribution in rats. J. Control. Release Off. J. Control. Release Soc..

[130] Mei B., Jiang H., Tjandra H., Strauss J., Chen Y., Liu T., Zhang X., Severs J., Newgern J., Chen J. (2010). Rational design of a fully active, long-acting PEGylated factor VIII for hemophilia A treatment. Blood.

[131] Xie D., Yao C., Wang L., Min W., Xu J., Xiao J., Huang M., Chen B., Liu B., Li X. (2010). An albumin-conjugated peptide exhibits potent anti-HIV activity and long in vivo half-life. Antimicrob. Agents Chemother..

[132] Liechty W.B., Peppas N.A. (2012). Expert opinion: Responsive polymer nanoparticles in cancer therapy. Eur. J. Pharm. Biopharm..

[133] Ganta S., Devalapally H., Shahiwala A., Amiji M. (2008). A review of stimuli-responsive nanocarriers for drug and gene delivery. J. Control. Release Off. J. Control. Release Soc..

[134] MacEwan S.R., Callahan D.J., Chilkoti A. (2010). Stimulus-responsive macromolecules and nanoparticles for cancer drug delivery. Nanomedicine.

[135] Mura S., Nicolas J., Couvreur P. (2013). Stimuli-responsive nanocarriers for drug delivery. Nat. Mater..

[136] Bennet D., Kim S. (2014). Polymer nanoparticles for smart drug delivery. Application of Nanotechnology in Drug Delivery.

[137] Shao P., Wang B., Yazhou W., Li J., Zhnag Y. (2011). The application of thermosensitive nanocarriers in controlled drug delivery. J. Nanomater..

[138] Kim J.-C., Kim M.-S., Kim J.-D. (1999). Temperature-sensitive releases from liposomes containing hydrophobically modified poly(N-isopropylacrylamide). Korean J. Chem. Eng..

[139] Kono K., Ozawa T., Yoshida T., Ozaki F., Ishizaka Y., Maruyama K., Kojima C., Harada A., Aoshima S. (2010). Highly temperature-sensitive liposomes based on a thermosensitive block copolymer for tumor-specific chemotherapy. Biomaterials.

[140] Karimi M., Sahandi Zangabad P., Ghasemi A., Amiri M., Bahrami M., Malekzad H., Ghahramanzadeh Asl H., Mahdieh Z., Bozorgomid M., Ghasemi A. (2016). Temperature-responsive smart nanocarriers for the delivery of therapeutic agents: Applications and recent advances. ASC Appl. Mater. Interfaces.

[141] Kokardekar R.R., Shah V., Mody H.R. (2012). PNIPAM poly (N-isopropylacrylamide): A thermosensitive “smart” polymer in novel drug delivery systems. Internet J. Med. Update.

[142] Pang C.-L., Tsai H.-M., Yang S.-J., Luo T.-Y., Lin C.-F., Lin W.-J., Shieh M.-J. (2011). Development of thermosensitive poly(n-isopropylacrylamide-co-((2-dimethylamino)ethyl methacrylate))-based nanoparticles for controlled drug release. Nanotechnology.

[143] Luo Y.-L., Yu W., Xu F., Zhang L.-L. (2012). Novel thermo-responsive self-assembly micelles from a double brush-shaped PNIPAM-g-(PA-b-PEG-b-PA)-g-PNIPAM block copolymer with PNIPAM polymers as side chains. J. Polym. Sci. Part Polym. Chem..

[144] Hamner K.L., Maye M.M. (2013). Thermal aggregation properties of nanoparticles modified with temperature sensitive copolymers. Langmuir.

[145] Tamarov K., Xu W., Osminkina L., Zinovyev S., Soininen P., Kurdyavtsev A., Gaydarova M., Närvänen A., Timoshenko V., Lehto V.-P. (2016). Temperature responsive porous silicon nanoparticles for cancer therapy-spatiotemporal triggering through infrared and radiofrequency electromagnetic heating. J. Control. Release.

[146] Han H.D., Shin B.C., Choi H.S. (2006). Doxorubicin-encapsulated thermosensitive liposomes modified with poly(N-isopropylacrylamide-co-acrylamide): Drug release behavior and stability in the presence of serum. Eur. J. Pharm. Biopharm..

[147] Shirakura T., Kelson T.J., Ray A., Malyarenko A.E., Kopelman R. (2014). Hydrogel nanoparticles with thermally controlled drug release. ACS Macro Lett..

[148] Kneidl B., Peller M., Winter G., Lindner L.H., Hossann M. (2014). Thermosensitive liposomal drug delivery systems: State of the art review. Int. J. Nanomed..

[149] Li J., Wang X., Zhang T., Wang C., Huang Z., Luo X., Deng Y. (2015). A review on phospholipids and their main applications in drug delivery systems. Asian J. Pharm. Sci..

[150] Ta T., Porter T.M. (2013). Thermosensetive liposomes for localized delivery and triggered release of chemotherapy. J. Control. Release.

[151] Wang Z.-Y., Zhang H., Yang Y., Xie X.-Y., Yang Y.-F., Li Z., Li Y., Gong W., Yu F.-L., Yang Z. (2016). Preparation, characterization, and efficacy of thermosensitive liposomes containing paclitaxel. Drug Deliv..

[152] May J.P., Ernsting M.J., Undzys E., Li S.-D. (2013). Thermosensitive liposomes for the delivery of gemcitabine and oxaliplatin to tumors. Mol. Pharm..

[153] Zeng C., Yu F., Yang Y., Cheng X., Liu Y., Zhang H., Zhao S., Yang Z., Li M., Li Z. (2016). Preparation and evaluation of oxaliplatin thermosensitive liposomes with rapid release and high stability. PLoS ONE.

[154] Stover T.C., Kim Y.S., Lowe T.L., Kester M. (2008). Thermoresponsive and biodegradable linear-dendritic nanoparticles for targeted and sustained release of a pro-apoptotic drug. Biomaterials.

[155] Yan B., Boyer J.-C., Habault D., Branda N.R., Zhao Y. (2012). Near infrared light triggered release of biomacromolecules from hydrogels loaded with upconversion nanoparticles. J. Am. Chem. Soc..

[156] Fomina N., Sankaranarayanan J., Almutairi A. (2012). Photochemical mechanisms of light-triggered release from nanocarriers. Adv. Drug Deliv. Rev..

[157] Alvarez-Lorenzo C., Bromberg L., Concheiro A. (2009). Light-sensitive intelligent drug delivery systems. Photochem. Photobiol..

[158] Kamaly N., Yameen B., Wu J., Farokhzad O.C. (2016). Degradable controlled-release polymers and polymeric nanoparticles: Mechanisms of controlling drug release. Chem. Rev..

[159] Son S., Shin E., Kim B.-S. (2014). Light-responsive micelles of spiropyran initiated hyperbranched polyglycerol for smart drug delivery. Biomacromolecules.

[160] Nishiyama N., Iriyama A., Jang W.-D., Miyata K., Itaka K., Inoue Y., Takahashi H., Yanagi Y., Tamaki Y., Koyama H. (2005). Light-induced gene transfer from packaged DNA enveloped in a dendrimeric photosensitizer. Nat. Mater..

[161] Rijcken C.J.F., Soga O., Hennink W.E., van Nostrum C.F. (2007). Triggered destabilisation of polymeric micelles and vesicles by changing polymers polarity: An attractive tool for drug delivery. J. Control. Release Off. J. Control. Release Soc..

[162] He J., Tong X., Zhao Y. (2009). Photoresponsive nanogels based on photocontrollable cross-links. Macromolecules.

[163] Paasonen L., Laaksonen T., Johans C., Yliperttula M., Kontturi K., Urtti A. (2007). Gold nanoparticles enable selective light-induced contents release from liposomes. J. Control. Release Off. J. Control. Release Soc..

[164] Chen X., Liu L., Jiang C. (2016). Charge-reversal nanoparticles: Novel targeted drug delivery carriers. Acta Pharm. Sin. B.

[165] Zhang R., Yao R., Ding B., Shen Y., Shui S., Wang L., Li Y., Yang X., Tao W. (2014). Fabrication of upconverting hybrid nanoparticles for near-infrared light triggered drug release. Adv. Mater. Sci. Eng..

[166] Volodkin D.V., Skirtach A.G., Möhwald H. (2009). Near-IR remote release from assemblies of liposomes and nanoparticles. Angew. Chem. Int. Ed..

[167] Jayakumar M.K.G., Idris N.M., Zhang Y. (2012). Remote activation of biomolecules in deep tissues using near-infrared-to-UV upconversion nanotransducers. Proc. Natl. Acad. Sci. USA.

[168] Zhang Z., Wang L., Wang J., Jiang X., Li X., Hu Z., Ji Y., Wu X., Ceh C. (2012). Mesoporous silica-coated gold nanorods as a light-mediated multifunctional theranostic platform for cancer treatment. Adv. Mater..

[169] Thambi T., Deepagan V.G., Yoon H.Y., Han H.S., Kim S.-H., Son S., Jo D.-G., Ahn C.-H., Suh Y.D., Kim K. (2014). Hypoxia-responsive polymeric nanoparticles for tumor-targeted drug delivery. Biomaterials.

[170] Kulkarni P., Haldar M.K., You S., Choi Y., Mallik S. (2016). Hypoxia-responsive polymersomes for drug delivery to hypoxic pancreatic cancer cells. Biomacromolecules.

[171] Lue Y., Aimmetti A.A., Langer R., Gu Z. (2016). Bioresponsive materials. Nat. Rev. Mater..

[172] Zhang R., Li Y., Zhang M., Tang Q., Zhang X. (2016). Hypoxia-responsive drug-drug conjugated nanoparticles for breast cancer synergistic therapy. Recent Adv..

[173] Kulkarni P., Haldar M.K., Katti P., Dawes C., You S., Choi Y., Mallik S. (2016). Hypoxia responsive, tumor penetrating lipid nanopyrticles for delivery of chemotherapeutics to pancreatic cancer cell spheroids. Bioconjug. Chem..

[174] Liu H., Zhang R., Niu Y., Qiao C., Weng J., Wang X., Xiao Z., Zhang X. (2015). Development of hypoxia triggered prodrug micelles as doxorubicin carriers for tumor therapy. RSC Adv..

[175] Aldea M., Florian I.A., Kacso G., Craciun L., Boca S., Soritau O., Florian I.S. (2016). Nanoparticles for targeting intratumoral hypoxia: Exploiting a potential weakness of glioblasoma. Pharm. Res..

[176] Qian C., Yu J., Chen Y., Hu Q., Xiao X., Sun W., Wan C., Feng P., Shen Q.-D., Gu Z. (2016). Light-activated hypoxia-responsive nanocarriers for enhanced anticancer therapy. Adv. Mater..

[177] Gao W., Chan J.M., Farokhzad O.C. (2010). pH-Responsive nanoparticles for drug delivery. Mol. Pharm..

[178] Shen Y., Tang H., Radosz M., Van Kirk E., Murdoch W.J. (2008). pH-Responsive nanoparticles for cancer drug delivery. Drug Delievery Systems.

[179] Chang G., Li C., Lu W., Ding J. (2010). N-Boc-histidine-capped PLGA-PEG-PLGA as a smart polymer for drug delivery sensitive to tumor extracellular pH. Macromol. Biosci..

[180] Ko J., Park K., Kim Y.-S., Kim M.S., Han J.K., Kim K., Park R.-W., Kim I.-S., Song H.K., Lee D.S. (2007). Tumoral acidic extracellular pH targeting of pH-responsive MPEG-poly(beta-amino ester) block copolymer micelles for cancer therapy. J. Control. Release Off. J. Control. Release Soc..

[181] Poon Z., Chang D., Zhao X., Hammond P.T. (2011). Layer-by-layer nanoparticles with a pH-sheddable layer for in vivo targeting of tumor hypoxia. ACS Nano.

[182] Tran T.H., Ramasamy T., Choi J.Y., Nguyen H.T., Pham T.T., Jeong J.-H., Ku S.K., Choi H.-G., Yong C.S., Kim J.O. (2015). Tumor-targeting, pH-sensitive nanoparticles for docetaxel delivery to drug-resistant cancer cells. Int. J. Nanomed..

[183] Meng F., Zhong Y., Cheng R., Deng C., Zhong Z. (2014). pH-sensitive polymeric nanoparticles for tumor-targeting doxorubicin delivery: Concept and recent advances. Nanomedicine.

[184] Heffernan M.J., Murthy N. (2005). Polyketal nanoparticles: A new pH-sensitive biodegradable drug delivery vehicle. Bioconjug. Chem..

[185] Qiu L., Hu Q., Cheng L., Li L., Tian C., Chen W., Chen Q., Hu W., Xu L., Yang J. (2016). cRGDyK modified pH responsive nanoparticles for specific intracellular delivery of doxorubicin. Acta Biomater..

[186] Kanamala M., Wilson W.R., Yang M., Palmer B.D., Wu Z. (2016). Mechanisms and biomaterials in pH-responsive tumour targeted drug delivery: A review. Biomaterials.

[187] Du J.-Z., Du X.-J., Mao C.-Q., Wang J. (2011). Tailor-made dual pH-sensitive polymer-doxorubicin nanoparticles for efficient anticancer drug delivery. J. Am. Chem. Soc..

[188] She W., Luo K., Zhang C., Wang G., Geng Y., Li L., He B., Gu Z. (2013). The potential of self-assembled, pH-responsive nanoparticles of mPEGylated peptide dendron-doxorubicin conjugates for cancer therapy. Biomaterials.

[189] He H., Chen S., Zhou J., Dou Y., Song L., Che L., Zhou X., Chen X., Jia Y., Zhang J. (2013). Cyclodextrin-derived pH-responsive nanoparticles for delivery of paclitaxel. Biomaterials.

[190] Aryal S., Hu C.-M.J., Zhang L. (2010). Polymer—Cisplatin conjugate nanoparticles for acid-responsive drug delivery. ACS Nano.

[191] Lee C.-H., Cheng S.-H., Huang I.-P., Souris J.S., Yang C.-S., Mou C.-Y., Lo L.-W. (2010). Intracellular pH-responsive mesoporous silica nanoparticles for the controlled release of anticancer chemotherapeutics. Angew. Chem. Int. Ed Engl..

[192] Zhao H., Duong H.H.P., Yung L.Y.L. (2010). Folate-conjugated polymer micelles with pH-triggered drug release properties. Macromol. Rapid Commun..

[193] Duan C., Gao J., Zhang D., Jia L., Liu Y., Zheng D., Liu G., Tian X., Wang F., Zhang Q. (2011). Galactose-decorated pH-responsive nanogels for hepatoma-targeted delivery of oridonin. Biomacromolecules.

[194] Fan L., Wu H., Zhang H., Li F., Yang T., Gu C., Yang Q. (2008). Novel super pH-sensitive nanoparticles responsive to tumor extracellular pH. Carbohydr. Polym..

[195] Wu X.L., Kim J.H., Koo H., Bae S.M., Shin H., Kim M.S., Lee B.-H., Park R.-W., Kim I.-S., Choi K. (2010). Tumor-targeting peptide conjugated pH-responsive micelles as a potential drug carrier for cancer therapy. Bioconjug. Chem..

[196] Oh K., Oh Y., Oh N., Kim K., Lee E. (2009). A smart flower-like polymeric micelle for pH-triggered anticancer drug release. Int. J. Pahrmaceutics.

[197] Filippov S., Hrubý M., Koňák Č., Macková H., Špírková M., Štěpánek P. (2008). Novel pH-responsive nanoparticles. Langmuir.

[198] Andrieu J. (2011). Specific Enzymatic Cleavage and Payload Release from Peptide-Based Hybrid Nanocapsules. Ph.D. Thesis.

[199] Park H.-K., Lee S.J., Oh J.-S., Lee S.-G., Jeong Y.-I., Lee H.C. (2015). Smart nanoparticles based on hyaluronic acid for redox-responsive and CD44 receptor-mediated targeting of tumor. Nanoscale Res. Lett..

[200] Zhao M., Biswas A., Hu B., Joo K.-I., Wang P., Gu Z., Tang Y. (2011). Redox-responsive nanocapsules for intracellular protein delivery. Biomaterials.

[201] Chuan X., Song Q., Lin J., Chen X., Zhang H., Dai W., He B., Wang X., Zhang Q. (2014). Novel free-paclitaxel-loaded redox-responsive nanoparticles based on a disulfide-linked poly(ethylene glycol)−drug conjugate for intracellular drug delivery: Synthesis, characterization, and antitumor activity In Vitro and In Vivo. Mol. Pharm..

[202] Gong H., Xie Z., Liu M., Sun H., Zhu H., Guo H. (2015). Research on redox-responsive mesoporous silica nanoparticles functionalized with PEG via a disulfide bond linker as drug carrier materials. Colloid Polym. Sci..

[203] Zhou Z., Tang J., Sun Q., Murdoch W.J., Shen Y. (2015). A multifunctional PEG–PLL drug conjugate forming redox-responsive nanoparticles for intracellular drug delivery. J. Mater. Chem. B.

[204] Luo Z., Cai K., Hu Y., Li J., Ding X., Zhang B., Xu D., Yang W., Liu P. (2012). Redox-responsive molecular nanoreservoirs for controlled intracellular anticancer drug delivery based on magnetic nanoparticles. Adv. Mater..

[205] Mejia-Ariza R., Kronig G.A., Huskens J. (2015). Size-controlled and redox-responsive supramolecular nanoparticles. Beilstein J. Org. Chem..

[206] Hu X.-Y., Chen Y., Liu Y. (2015). Redox-responsive supramolecular nanoparticles based on amphiphilic sulfonatocalixarene and selenocystamine dihydrochloride. Chin. Chem. Lett..

[207] Wen H., Li Y. (2014). Redox sensitive nanoparticles with disulfide bond linked sheddable shell for intracellular drug delivery. Med. Chem..

[208] Song N., Liu W., Tu Q., Liu R., Zhang Y., Wang J. (2011). Preparation and in vitro properties of redox-responsive polymeric nanoparticles for paclitaxel delivery. Colloids Surf. B Biointerfaces.

[209] Xu J., Singh A., Amiji M.M. (2014). Redox-responsive targeted gelatin nanoparticles for delivery of combination wt-p53 expressing plasmid DNA and gemcitabine in the treatment of pancreatic cancer. BMC Cancer.

[210] Alvarez-Berríos M.P., Vivero-Escoto J.L. (2016). In Vitro evaluation of folic acid-conjugated redox-responsive mesoporous silica nanoparticles for the delivery of cisplatin. Int. J. Nanomed..

[211] de la Rica R., Aili D., Stevens M.M. (2012). Enzyme-responsive nanoparticles for drug release and diagnostics. Adv. Drug Deliv. Rev..

[212] Callmann C.E. (2015). Therapeutic enzyme-responsive nanoparticles for targeted delivery and accumulation in tumors. Adv. Mater..

[213] Nguyen M.M., Carlini A.S., Chien M.-P., Sonnenberg S., Luo C., Braden R.L., Osborn K.G., Li Y., Gianneschi N.C., Christman K.L. (2015). Enzyme-responsive nanoparticles for targeted accumulation and prolonged retention in heart tissue after myocardial infarction. Adv. Mater..

[214] Insua I., Liamas E., Zhang Z., Peacock A.F., Krachler A.M., Fernandez-Trillo F. (2016). Enzyme-responsive polyion complex (PIC) nanoparticles for the targeted delivery of antimicrobial polymers. Polym. Chem..

[215] Hu Q., Katti P., Gu Z. (2014). Enzyme-responsive destabilization of stabilized plasmid-lipid nanoparticles as an efficient gene delivery. Nanoscale.

[216] Choi K.Y., Swierczewska M., Lee S., Chen X. (2012). Protease-activated drug development. Theranostics.

[217] Lee S.J., Jeong Y.-I., Park H.-K., Kang D.H., Oh J.-S., Lee S.-G., Lee H.C. (2015). Enzyme-responsive doxorubicin release from dendrimer nanoparticles for anticancer drug delivery. Int. J. Nanomed..

[218] Yang Y., Aw J., Chen K., Liu F., Padmanabhan P., Hou Y., Cheng Z., Xing B. (2011). Enzyme-responsive multifunctional magnetic nanoparticles for tumor intracellular drug delivery and imaging. Chem. Asian J..

[219] Hou X.-F., Chen Y., Liu Y. (2015). Enzyme-responsive protein/polysaccharide supramolecular nanoparticles. Soft Matter.

[220] Bernardos A., Mondragon L., Aznar E., Marcos M.D., Martinez-Mañez R., Sancenon F., Soto J., Barat J.M., Perez-Paya E., Guillem C. (2010). Enzyme-responsive intracellular controlled release using nanometric silica mesoporous supports capped with “saccharides”. ACS Nano.

[221] Patel K., Angelos S., Dichtel W.R., Coskun A., Yang Y.-W., Zink J.I., Stoddart J.F. (2008). Enzyme-responsive snap-top covered silica nanocontainers. J. Am. Chem. Soc..

[222] Basel M.T., Shrestha T.B., Troyer D.L., Bossmann S.H. (2011). Protease-sensitive, polymer-caged liposomes: A method for making highly targeted liposomes using triggered release. ACS Nano.

[223] Samanta D., Hosseini-Nassab N., Zare R.N. (2016). Electroresponsive nanoparticles for drug delivery on demand. Nanoscale.

[224] Ge J., Neofytou E., Cahill T.J., Beygui R.E., Zare R.N. (2012). Drug release from electric-field-responsive nanoparticles. ACS Nano.

[225] Li F., Zhu Y., Wang Y. (2014). Dual-responsive drug delivery system with real time tunable release behavior. Microporous Mesoporous Mater..

[226] Yan Q., Yuan J., Cai Z., Xin Y., Kang Y., Yin Y. (2010). Voltage-responsive vesicles based on orthogonal assembly of two homopolymers. J. Am. Chem. Soc..

[227] Ying X., Wang Y., Liang J., Yue J., Xu C., Lu L., Xu Z., Gao J., Du Y., Chen Z. (2014). Angiopep-conjugated electro-responsive hydrogel nanoparticles: Therapeutic potential for epilepsy. Angew. Chem..

[228] Wang Y., Ying X., Chen L., Liu Y., Wang Y., Liang J., Xu C., Guo Y., Wang S., Hu W. (2016). Electroresponsive nanoparticles improve antiseizure effect of phenytoin in generalized tonic-clonic seizures. Neurotherapeutics.

[229] Pankhurst Q.A., Connolly J., Jones S.K., Dobson J. (2003). Applications of magnetic nanoparticles in biomedicine. J. Phys. D Appl. Phys..

[230] Bucak S., Yavuztürk B., Sezer A.D. (2012). Magnetic nanoparticles: Synthesis, surface modifications and application in drug delivery. Recent Advances in Nivel Drug Carrier System.

[231] McBain S.C., Yiu H.H., Dobson J. (2008). Magnetic nanoparticles for gene and drug delivery. Int. J. Nanomed..

[232] Estelrich J., Escribano E., Queralt J., Busquets M.A. (2015). Iron oxide nanoparticles for magnetically-guided and magnetically-responsive drug delivery. Int. J. Mol. Sci..

[233] Qin J., Asempah I., Laurent S., Fornara A., Muller R.N., Muhammed M. (2009). Injectable superparamagnetic ferrogels for controlled release of hydrophobic drugs. Adv. Mater..

[234] Kumar C.S.S.R., Mohammad F. (2011). Magnetic nanomaterials for hyperthermia-based therapy and controlled drug delivery. Adv. Drug Deliv. Rev..

[235] Monnier C.A., Burnand D., Rothen-Rutishauser B., Lattuada M., Petri-Fink A. (2014). Magnetoliposomes: Opportunities and challenges. Eur. J. Nanomed..

[236] Kong S.D., Zhang W., Lee J.H., Brammer K., Lal R., Karin M., Jin S. (2010). Magnetically vectored nanocapsules for tumor penetration and remotely switchable on-demand drug release. Nano Lett..

[237] Liu T.-Y., Hu S.-H., Liu K.-H., Shaiu R.-S., Liu D.-M., Chen S.-Y. (2008). Instantaneous drug delivery of magnetic/thermally sensitive nanospheres by a high-frequency magnetic field. Langmuir ACS J. Surf. Colloids.

[238] Giri S., Trewyn B.G., Stellmaker M.P., Lin V.S.-Y. (2005). Stimuli-responsive controlled-release delivery system based on mesoporous silica nanorods capped with magnetic nanoparticles. Angew. Chem. Int. Ed Engl..

[239] Ruiz-Hernández E., Baeza A., Vallet-Regí M. (2011). Smart drug delivery through DNA/magnetic nanoparticle gates. ACS Nano.

[240] Hayashi K., Ono K., Suzuki H., Sawada M., Moriya M., Sakamoto W., Yogo T. (2010). High-frequency, magnetic-field-responsive drug release from magnetic nanoparticle/organic hybrid based on hyperthermic effect. ACS Appl. Mater. Interfaces.

[241] Tan S., Wang G. (2017). Redox-responsive and pH-sensitive nanoparticles enhanced stability and anticancer ability of erlotinib to treat lung cancer In Vivo. Drug Des. Devel. Ther..

[242] Wang S., Zhang S., Liu J., Liu Z., Su L., Wang H., Chang J. (2014). pH- and reduction-responsive polymeric lipid vesicles for enhanced tumor cellular internalization and triggered drug release. ACS Appl. Mater. Interfaces.

[243] Unsoy G., Khodadust R., Yalcin S., Mutlu P., Gunduz U. (2014). Synthesis of Doxorubicin loaded magnetic chitosan nanoparticles for pH responsive targeted drug delivery. Eur. J. Pharm. Sci. Off. J. Eur. Fed. Pharm. Sci..

[244] Klaikherd A., Nagamani C., Thayumanavan S. (2009). Multi-stimuli sensitive amphiphilic block copolymer assemblies. J. Am. Chem. Soc..

[245] Cao Z., Wu H., Dong J., Wang G. (2014). Quadruple-stimuli-sensitive polymeric nanocarriers for controlled release under combined stimulation. Macromolecules.

[246] Wen J., Sun S. (2017). Recent advances of multi-stimuli-responsive drug delivery systems for cancer therapy. Curr. Trends Biomed. Eng. Biosci..

[247] Strebhardt K., Ullrich A. (2008). Paul Ehrlich’s magic bullet concept: 100 years of progress. Nat. Rev. Cancer.

[248] Shi J., Kantoff P.W., Wooster R., Farokhzad O.C. (2017). Cancer nanomedicine: Progress, challenges and opportunities. Nat. Rev. Cancer.

[249] Weissig V., Guzman-Villanueva D. (2015). Nanopharmaceuticals (part 2): Products in the pipeline. Int. J. Nanomed..

[250] Lammers T., Kiessling F., Ashford M., Hennink W., Crommelin D., Storm G. (2016). Cancer nanomedicine: Is targeting our target?. Nat. Rev. Mater..

[251] Sun Q., Ojha T., Kiessling F., Lammers T., Shi Y. (2017). Enhancing tumor penetration of nanomedicines. Biomacromolecules.

[252] Dasgupta A., Liu M., Ojha T., Storm G., Kiessling F., Lammers T. (2016). Ultrasound-mediated drug delivery to the brain: Principles, progress and prospects. Drug Discov. Today Technol..

[253] Nie S. (2010). Understanding and overcoming major barriers in cancer nanomedicine. Nanomedicine.

[254] Tammam S., Azzazy H.M., Lamprecht A. (2015). Biodegradable particulate carrier formulation and tuning for targeted drug delivery. J. Biomed. Nanotechnol..

[255] Li S.-D., Huang L. (2009). Nanoparticles evading the reticuloendotherlial system: Role of the supported bilayer. Biochim. Biophys. Acta BBA Biomembr..

[256] Pearson R.M., Juettner V.V., Hong S. (2014). Biomolecular corona on nanoparticles: A survey of recent literature and its implications in targeted drug delivery. Front. Chem..

[257] Guo S., Huang L. (2011). Nanoparticles escaping RES and endosome: Challenges for siRNA delivery for cancer therapy. J. Nanomater..

[258] Aoyama M., Hata K., Higashisaka K., Nagano K., Yoshioka Y., Tsutsumi Y. (2016). Clusterin in the protein corona plays a key role in the stealth effect of nanoparticles against phagocytes. Biochem. Biophys. Res. Commun..

[259] Blanco E., Shen H., Ferrari M. (2015). Principles of nanoparticle design for overcoming biological barriers to drug delivery. Nat. Biotechnol..

[260] Rao L., Xu J.-H., Cai B., Liu H., Li M., Jia Y., Xiao L., Guo S.-S., Liu W., Zhao X.-Z. (2016). Synthetic nanoparticles camouflaged with biomimetic erythrocyte membranes for reduced reticuloendothelial system uptake. Nanotechnology.

[261] Magaña I.B., Yendluri R.B., Adhikari P., Goodrich G.P., Schwartz J.A., Sherer E.A., O’Neal D.P. (2015). Suppression of the reticuloendothelial system using λ-carrageenan to prolong the circulation of gold nanoparticles. Ther. Deliv..

[262] Elzoghby A.O., Samy W.M., Elgindy N.A. (2012). Albumin-based nanoparticles as potential controlled release drug delivery systems. J. Control. Release Off. J. Control. Release Soc..

[263] Lim S.I., Minzuta Y., Takasu A., Hahn Y.S., King Y.H., Kwon I. (2013). Site-specific fatty acid-conjugation to prolong protein half-life In Vivo. J. Control. Release.

[264] Bazak R., Houri M., Achy S.E., Hussein W., Refaat T. (2014). Passive targeting of nanoparticles to cancer: A comprehensive review of the literature. Mol. Clin. Oncol..

[265] Talekar M., Tran T.-H., Amiji M. (2015). Translational nano-medicines: Targeted therapeutic delivery for cancer and inflammatory diseases. AAPS J..

[266] Ulbrich W., Lamprecht A. (2010). Targeted drug-delivery approaches by nanoparticulate carriers in the therapy of inflammatory diseases. J. R. Soc. Interface.

[267] Look M., Stern E., Wang Q.A., DiPlacido L.D., Kashgarian M., Craft J., Fahmy T.M. (2013). Nanogel-based delivery of mycophenolic acid ameliorates systemic lupus erythematosus in mice. J. Clin. Investig..

[268] Lundy D.J., Chen K.-H., Toh E.K.-W., Hsieh P.C.-H. (2016). Distribution of systemically administered nanoparticles reveals a size-dependent effect immediately following cardiac ischaemia-reperfusion injury. Sci. Rep..

[269] Goyal K., Koul V., Singh Y., Anand A. (2014). Targeted drug delivery to central nervous system (CNS) for the treatment of neurodegenerative disorders: Trends and advances. Cent. Nerv. Syst. Agents Med. Chem..

[270] Olivier J.C., Fenart L., Chauvet R., Pariat C., Cecchelli R., Couet W. (1999). Indirect evidence that drug brain targeting using polysorbate 80-coated polybutylcyanoacrylate nanoparticles is related to toxicity. Pharm. Res..

[271] Xu L., Dan M., Shao A., Cheng X., Zhang C., Yokel R.A., Takemura T., Hanagata N., Niwa M., Watanabe D. (2015). Silver nanoparticles induce tight junction disruption and astrocyte neurotoxicity in a rat blood–brain barrier primary triple coculture model. Int. J. Nanomed..

[272] Georgieva J.V., Kalicharan D., Couraud P.-O., Romero I.A., Weksler B., Hoekstra D., Zuhorn I.S. (2011). Surface characteristics of nanoparticles determine their intracellular fate in and processing by human blood–brain barrier endothelial cells In Vitro. Mol. Ther..

[273] Saraiva C., Praça C., Ferreira R., Santos T., Ferreira L., Bernardino L. (2016). Nanoparticle-mediated brain drug delivery: Overcoming blood-brain barrier to treat neurodegenerative diseases. J. Control. Release Off. J. Control. Release Soc..

[274] Singh M.S., Tammam S.N., Shetab Boushehri M.A., Lamprecht A. (2017). MDR in cancer: Addressing the underlying cellular alterations with the use of nanocarriers. Pharmacol. Res..

[275] Nieto Montesinos R., Béduneau A., Pellequer Y., Lamprecht A. (2012). Delivery of P-glycoprotein substrates using chemosensitizers and nanotechnology for selective and efficient therapeutic outcomes. J. Control. Release.

[276] Williams R.M., Jaimes E.A., Heller D.A. (2016). Nanomedicines for kidney diseases. Kidney Int..

[277] Williams R.M., Shah J., Ng B.D., Minton D.R., Gudas L.J., Park C.Y., Heller D.A. (2015). Mesoscale nanoparticles selectively target the renal proximal tubule epithelium. Nano Lett..

[278] Williams R.M., Shah J., Tian H.S., Chen X., Geissmann F., Jaimes E.A., Heller D.A. (2018). Selective nanoparticle targeting of the renal tubules. Hypertens. Dallas Tex 1979.

[279] Nair A.V., Keliher E.J., Core A.B., Brown D., Weissleder R. (2015). Characterizing the interactions of organic nanoparticles with renal epithelial cells in vivo. ACS Nano.

[280] Bennett K.M., Zhou H., Sumner J.P., Dodd S.J., Bouraoud N., Doi K., Star R.A., Koretsky A.P. (2008). MRI of the basement membrane using charged nanoparticles as contrast agents. Magn. Reson. Med..

[281] Torres Andón F., Alonso M.J. (2015). Nanomedicine and cancer immunotherapy—Targeting immunosuppressive cells. J. Drug Target..

[282] Mishra H., Mishra D., Mishra P.K., Nahar M., Dubey V., Jain N.K. (2010). Evaluation of solid lipid nanoparticles as carriers for delivery of hepatitis B surface antigen for vaccination using subcutaneous route. J. Pharm. Pharm. Sci..

[283] Singh Y., Srinivas A., Gangwar M., Meher J.G., Misra-Bhattacharya S., Chourasia M.K. (2016). Subcutaneously administered ultrafine PLGA nanoparticles containing doxycycline hydrochloride target lymphatic filarial parasites. Mol. Pharm..

[284] Yu X., Trase I., Ren M., Duval K., Guo X., Chen Z. (2016). Design of nanoparticle-based carriers for targeted drug delivery. J. Nanomater..

[285] Chen W.C., Zhang A.X., Li S.-D. (2012). Limitations and niches of the active targeting approach for nanoparticle drug delivery. Eur. J. Nanomed..

[286] Arruebo M., Valladares M., González-Fernández Á. (2009). Antibody-conjugated nanoparticles for biomedical applications. J. Nanomater..

[287] Richards D.A., Maruani A., Chudasama V. (2016). Antibody fragments as nanoparticle targeting ligands: A step in the right direction. Chem. Sci..

[288] Kennedy P.J., Perreira I., Ferreira D., Nestor M., Oliveira C., Granja P.L., Sarmento B. (2018). Impact of surfactants on the target recognition of Fab-conjugated PLGA nanoparticles. Eur. J. Pharm. Biopharm..

[289] Rezaei G., Habibi-Anbouhi M., Mahmoudi M., Azadmanesh K., Moradi-Kalbolandi S., Behdani M., Ghazizadeh L., Abolhassani M., Shokrgozar M.A. (2017). Development of anti-CD47 single-chain variable fragment targeted magnetic nanoparticles for treatment of human bladder cancer. Nanomedicine.

[290] Hu C.-M.J., Kaushal S., Tran Cao H.S., Aryal S., Sartor M., Esener S., Bouvet M., Zhang L. (2010). Half-antibody functionalized lipid-polymer hybrid nanoparticles for targeted drug delivery to carcinoembryonic antigen presenting pancreatic cancer cells. Mol. Pharm..

[291] Girgis M.D., Federman N., Rochefort M.M., McCabe K.E., Wu A.M., Nagy J.O., Denny C., Tomlinson J.S. (2013). An engineered anti-CA19-9 cys-diabody for positron emission tomography imaging of pancreatic cancer and targeting of polymerized liposomal nanoparticles. J. Surg. Res..

[292] Kontermann R. (2012). Dual targeting strategies with bispecific antibodies. mAbs.

[293] Pietersz G.A., Wang X., Yap M.L., Lim B., Peter K. (2017). Therapeutic targeting in nanomedicine: The future lies in recombinant antibodies. Nanomedicine.

[294] Nygren P.-A., Skerra A. (2004). Binding proteins from alternative scaffolds. J. Immunol. Methods.

[295] Löfblom J., Feldwisch J., Tolmachev V., Carlsson J., Ståhl S., Frejd F.Y. (2010). Affibody molecules: Engineered proteins for therapeutic, diagnostic and biotechnological applications. FEBS Lett..

[296] Ståhl S., Gräslund T., Eriksson Karlström A., Frejd F.Y., Nygren P.-Å., Löfblom J. (2017). Affibody molecules in biotechnological and medical applications. Trends Biotechnol..

[297] Satpathy M., Wang L., Zielinski R., Qian W., Lipowska M., Capala J., Lee G.Y., Xu H., Wang Y.A., Mao H. (2014). Active targeting using HER-2-affibody-conjugated nanoparticles enabled sensitive and specific imaging of orthotopic HER-2 positive ovarian tumors. Small Weinh. Bergstr. Ger..

[298] Alexis F., Basto P., Levy-Nissenbaum E., Radovic-Moreno A.F., Zhang L., Pridgen E., Wang A.Z., Marein S.L., Westerhof K., Molnar L.K. (2008). HER-2-targeted nanoparticle-affibody bioconjugates for cancer therapy. ChemMedChem.

[299] Beuttler J., Rothdiener M., Müller D., Frejd F.Y., Kontermann R.E. (2009). Targeting of epidermal growth factor receptor (EGFR)-expressing tumor cells with sterically stabilized affibody liposomes (SAL). Bioconjug. Chem..

[300] Tiede C., Bedford R., Heseltine S.J., Smith G., Wijetunga I., Ross R., AlQallaf D., Roberts A.P., Balls A., Curd A. (2017). Affimer proteins are versatile and renewable affinity reagents. eLife.

[301] Khaled Y.S., Shamsuddin S., Tiernan J., McPherson M., Hughes T., Millner P., Jayne D.G. (2018). Theranostic CEA-Affimer functionalised silica nanoparticles allow specific in vitro fluorescent imaging of colorectal cancer cells. Eur. J. Surg. Oncol..

[302] Rawlings A.E., Bramble J.P., Tang A.A.S., Somner L.A., Monnington A.E., Cooke D.J., McPherson M.J., Tomlinson D.C., Staniland S.S. (2015). Phage display selected magnetite interacting Adhirons for shape controlled nanoparticle synthesis. Chem. Sci..

[303] Chiu H.-Y., Deng W., Engelke H., Helma J., Leonhardt H., Bein T. (2016). Intracellular chromobody delivery by mesoporous silica nanoparticles for antigen targeting and visualization in real time. Sci. Rep..

[304] Edelstein J. (2014). Fabrication of Protein Scaffold-Nanoparticle Conjugates for Targeted Cancer Therapeutics.

[305] Deyev S., Proshkina G., Ryabova A., Tavanti F., Menziani M.C., Eidelshtein G., Avishai G., Kotlyar A. (2017). Synthesis, characterization, and selective delivery of DARPin-gold nanoparticle conjugates to cancer cells. Bioconjug. Chem..

[306] Lee J.-J., Kang J.A., Ryu Y., Han S.-S., Nam Y.R., Rho J.K., Choi D.S., Kang S.-W., Lee D.-E., Kim H.-S. (2017). Genetically engineered and self-assembled oncolytic protein nanoparticles for targeted cancer therapy. Biomaterials.

[307] Wang A.Z., Gu F., Zhang L., Chan J.M., Radovic-Moreno A., Shaikh M.R., Farokhzad O.C. (2008). Biofunctionalized targeted nanoparticles for therapeutic applications. Expert Opin. Biol. Ther..

[308] Nasongkla N., Shuai X., Ai H., Weinberg B.D., Pink J., Boothman D.A., Gao J. (2004). cRGD-functionalized polymer micelles for targeted doxorubicin delivery. Angew. Chem. Int. Ed Engl..

[309] Yu X., Song Y., Di Y., He H., Fu D., Jin C. (2016). Enhanced tumor targeting of cRGD peptide-conjugated albumin nanoparticles in the BxPC-3 cell line. Sci. Rep..

[310] Danhier F., Pourcelle V., Marchand-Brynaert J., Jérôme C., Feron O., Préat V. (2012). Targeting of tumor endothelium by RGD-grafted PLGA-nanoparticles. Methods Enzymol..

[311] Endo-Takahashi Y., Ooaku K., Ishida K., Suzuki R., Maruyama K., Negishi Y. (2016). Preparation of Angiopep-2 peptide-modified bubble liposomes for delivery to the brain. Biol. Pharm. Bull..

[312] Chen G.-J., Su Y.-Z., Hsu C., Lo Y.-L., Huang S.-J., Ke J.-H., Kuo Y.-C., Wang L.-F. (2014). Angiopep-pluronic F127-conjugated superparamagnetic iron oxide nanoparticles as nanotheranostic agents for BBB targeting. J. Mater. Chem. B.

[313] Bidwell G.L., Mahdi F., Shao Q., Logue O.C., Waller J.P., Reese C., Chade A.R. (2017). A kidney-selective biopolymer for targeted drug delivery. Am. J. Physiol. Ren. Physiol..

[314] Geng Q., Sun X., Gong T., Zhang Z.-R. (2012). Peptide-drug conjugate linked via a disulfide bond for kidney targeted drug delivery. Bioconjug. Chem..

[315] Tammam S.N., Azzazy H.M.E., Breitinger H.G., Lamprecht A. (2015). Chitosan nanoparticles for nuclear targeting: The effect of nanoparticle size and nuclear localization sequence density. Mol. Pharm..

[316] Zhong Y., Meng F., Deng C., Zhong Z. (2014). Ligand-directed active tumor-targeting polymeric nanoparticles for cancer chemotherapy. Biomacromolecules.

[317] Bruschi M.L. (2019). Lectins and nanostructured drug delivery systems. Curr. Drug Deliv..

[318] Iskratsch T., Braun A., Paschinger K., Wilson I.B.H. (2009). Specificity analysis of lectins and antibodies using remodeled glycoproteins. Anal. Biochem..

[319] Hirabayashi J., Arai R. (2019). Lectin engineering: The possible and the actual. Interface Focus.

[320] Li J., Wu H., Hong J., Xu X., Yang H., Wu B., Wang Y., Zhu J., Lai R., Jiang X. (2008). Odorranalectin is a small peptide lectin with potential for drug delivery and targeting. PLoS ONE.

[321] Vineethkumar T.V., Shyla G., George S. (2018). Smallest lectin-like peptide identified from the skin secretion of an endemic frog, Hydrophylax bahuvistara. Acta Biol. Hung..

[322] Smart J.D. (2004). Lectin-mediated drug delivery in the oral cavity. Adv. Drug Deliv. Rev..

[323] Moulari B., Béduneau A., Pellequer Y., Lamprecht A. (2014). Lectin-decorated nanoparticles enhance binding to the inflamed tissue in experimental colitis. J. Control. Release Off. J. Control. Release Soc..

[324] Surti N., Misra A. (2008). Wheat germ agglutinin-conjugated nanoparticles for sustained cellular and lung delivery of budesonide. Drug Deliv..

[325] Nicholls T.J., Green K.L., Rogers D.J., Cook J.D., Wolowacz S., Smart J.D. (1996). Lectins in ocular drug delivery: An investigation of lectin binding sites on the corneal and conjunctival surfaces. Int. J. Pharm..

[326] Gao X., Tao W., Lu W., Zhang Q., Zhang Y., Jiang X., Fu S. (2006). Lectin-conjugated PEG–PLA nanoparticles: Preparation and brain delivery after intranasal administration. Biomaterials.

[327] Bies C., Lehr C.-M., Woodley J.F. (2004). Lectin-mediated drug targeting: History and applications. Adv. Drug Deliv. Rev..

[328] Poiroux G., Barre A., van Damme E.J.M., Benoist H., Rougé P. (2017). Plant lectins targeting O-glycans at the cell surface as tools for cancer diagnosis, prognosis and therapy. Int. J. Mol. Sci..

[329] Martínez-Carmona M., Lozano D., Colilla M., Vallet-Regí M. (2018). Lectin-conjugated pH-responsive mesoporous silica nanoparticles for targeted bone cancer treatment. Acta Biomater..

[330] Singh A., Dilnawaz F., Sahoo S.K. (2011). Long circulating lectin conjugated paclitaxel loaded magnetic nanoparticles: A new theranostic avenue for leukemia therapy. PLoS ONE.

[331] Obaid G., Chambrier I., Cook M.J., Russell D.A. (2012). Targeting the oncofetal Thomsen-Friedenreich disaccharide using jacalin-PEG phthalocyanine gold nanoparticles for photodynamic cancer therapy. Angew. Chem. Int. Ed Engl..

[332] Obaid G., Chambrier I., Cook M.J., Russell D.A. (2015). Cancer targeting with biomolecules: A comparative study of photodynamic therapy efficacy using antibody or lectin conjugated phthalocyanine-PEG gold nanoparticles. Photochem. Photobiol. Sci. Off. J. Eur. Photochem. Assoc. Eur. Soc. Photobiol..

[333] Coulibaly F.S., Youan B.-B.C. (2017). Current status of lectin-based cancer diagnosis and therapy. AIMS Mol. Sci..

[334] Lord J.M. (1987). The use of cytotoxic plant lectins in cancer therapy. Plant Physiol..

[335] Kröger A.P.P., Komil M.I., Hamelmann N.M., Juan A., Stenzel M.H., Paulusse J.M.J. (2019). Glucose single-chain polymer nanoparticles for cellular targeting. ACS Macro Lett..

[336] Carrillo-Conde B., Song E.H., Chavez-Santoscoy A., Phanse Y., Ramer-Tait A.E., Pohl N.L.B., Wannemuehler M.J., Bellaire B.H., Narasimhan B. (2011). Mannose-functionalized “pathogen-like” polyanhydride nanoparticles target C-type lectin receptors on dendritic cells. Mol. Pharm..

[337] Xia Y., Zhong J., Zhao M., Tang Y., Han N., Hua L., Xu T., Wang C., Zhu B. (2019). Galactose-modified selenium nanoparticles for targeted delivery of doxorubicin to hepatocellular carcinoma. Drug Deliv..

[338] Zhang Y., Chan J.W., Moretti A., Uhrich K.E. (2015). Designing polymers with sugar-based advantages for bioactive delivery applications. J. Control. Release Off. J. Control. Release Soc..

[339] Kikkeri R., Lepenies B., Adibekian A., Laurino P., Seeberger P.H. (2009). In Vitro imaging and In Vivo liver targeting with carbohydrate capped quantum dots. J. Am. Chem. Soc..

[340] Ahmed M., Narain R. (2015). Carbohydrate-based materials for targeted delivery of drugs and genes to the liver. Nanomedicine.

[341] Pranatharthiharan S., Patel M.D., Malshe V.C., Pujari V., Gorakshakar A., Madkaikar M., Ghosh K., Devarajan P.V. (2017). Asialoglycoprotein receptor targeted delivery of doxorubicin nanoparticles for hepatocellular carcinoma. Drug Deliv..

[342] Kou L., Bhutia Y.D., Yao Q., He Z., Sun J., Ganapathy V. (2018). Transporter-guided delivery of nanoparticles to improve drug permeation across cellular barriers and drug exposure to selective cell types. Front. Pharmacol..

[343] Qin Y., Fan W., Chen H., Yao N., Tang W., Tang J., Yuan W., Kuai R., Zhang Z., Wu Y. (2010). In Vitro and In Vivo investigation of glucose-mediated brain-targeting liposomes. J. Drug Target..

[344] Dufes C., Gailard F., Uchegbu I., Schätzlein A., Olivier J.-C., Muller J.-M. (2004). Glucose-targeted niosomes deliver vasoactive intestinal peptide (VIP) to the brain. Int. J. Pharm..

[345] Buchanan M.K., Needham C.N., Neill N.E., White M.C., Kelly C.B., Mastro-Kishton K., Chauvigne-Hines L.M., Goodwin T.J., McIver A.L., Bartolotti L.J. (2017). Glycoconjugated site-selective DNA-methylating agent targeting glucose transporters on glioma cells. Biochemistry.

[346] Sztandera K., Działak P., Marcinkowska M., Stańczyk M., Gorzkiewicz M., Janaszewska A., Klajnert-Maculewicz B. (2019). Sugar modification enhances cytotoxic activity of PAMAM-doxorubicin conjugate in glucose-deprived MCF-7 cells—Possible role of GLUT1 transporter. Pharm. Res..

[347] Park J.-H., Cho H.-J., Kim D.-D. (2017). Poly((D,L)lactic-glycolic)acid–star glucose nanoparticles for glucose transporter and hypoglycemia-mediated tumor targeting. Int. J. Nanomed..

[348] Chen F., Huang G., Huang H. (2020). Sugar ligand-mediated drug delivery. Future Med. Chem..

[349] De Coen R., Vanparijs N., Risseeuw M.D.P., Lybaert L., Louage B., De Koker S., Kumar V., Grooten J., Taylor L., Ayres N. (2016). pH-degradable mannosylated nanogels for dendritic cell targeting. Biomacromolecules.

[350] Asthana G.S., Asthana A., Kohli D.V., Vyas S.P. (2014). Mannosylated chitosan nanoparticles for delivery of antisense oligonucleotides for macrophage targeting. BioMed Res. Int..

[351] Apostolopoulos V., Thalhammer T., Tzakos A.G., Stojanovska L. (2013). Targeting antigens to dendritic cell receptors for vaccine development. J. Drug Deliv..

[352] Hockl P.F., Wolosiuk A., Pérez-Sáez J.M., Bordoni A.V., Croci D.O., Toum-Terrones Y., Soler-Illia G.J.A.A., Rabinovich G.A. (2016). Glyco-nano-oncology: Novel therapeutic opportunities by combining small and sweet. Pharmacol. Res..

[353] Jain A., Agarwal A., Majumder S., Lariya N., Khaya A., Agrawal H., Majumdar S., Agrawal G.P. (2010). Mannosylated solid lipid nanoparticles as vectors for site-specific delivery of an anti-cancer drug. J. Control. Release Off. J. Control. Release Soc..

[354] Jo H., Ban C. (2016). Aptamer–nanoparticle complexes as powerful diagnostic and therapeutic tools. Exp. Mol. Med..

[355] Aghanejad A., Babamiri H., Adibkia K., Barar J., Omidi Y. (2018). Mucin-1 aptamer-armed superparamagnetic iron oxide nanoparticles for targeted delivery of doxorubicin to breast cancer cells. BioImpacts.

[356] Aravind A., Varghese S.H., Veeranarayanan S., Mathew A., Nagaoka Y., Iwai S., Fukuda T., Hasumura T., Yoshida Y., Maekawa T. (2012). Aptamer-labeled PLGA nanoparticles for targeting cancer cells. Cancer Nanotechnol..

[357] Weigum S., McIvor E., Munoz C., Feng R., Cantu T., Walsh K., Betancourt T. (2016). Targeted therapy of hepatocellular carcinoma with aptamer-functionalized biodegradable nanoparticles. J. Nanoparticle Res..

[358] Liu J., Wei T., Zhao J., Huang Y., Deng H., Kumar A., Wang C., Liang Z., Ma X., Liang X.-J. (2016). Multifunctional aptamer-based nanoparticles for targeted drug delivery to circumvent cancer resistance. Biomaterials.

[359] Liang C., Guo B., Wu H., Shao N., Li D., Liu J., Dang L., Wang C., Li H., Li S. (2015). Aptamer-functionalized lipid nanoparticles targeting osteoblasts as a novel RNA interference-based bone anabolic strategy. Nat. Med..

[360] Yu L., Hu Y., Duan J., Yang X.-D. (2015). A novel approach of targeted immunotherapy against adenocarcinoma cells with nanoparticles modified by CD16 and MUC1 aptamers. J. Nanomater..

[361] Zhang P., Ye J., Liu E., Sun L., Zhang J., Lee S.-J., Gong J., He H., Yang V.C. (2017). Aptamer-coded DNA nanoparticles for targeted doxorubicin delivery using pH-sensitive spacer. Front. Chem. Sci. Eng..

[362] Catuogno S., Esposito C.L., de Franciscis V. (2016). Aptamer-mediated targeted delivery of therapeutics: An update. Pharmaceuticals.

[363] Farokhzad O.C., Karp J.M., Langer R. (2006). Nanoparticle-aptamer bioconjugates for cancer targeting. Expert Opin. Drug Deliv..

[364] Dixit S., Novak T., Miller K., Zhu Y., Kenney M.E., Broome A.-M. (2015). Transferrin receptor-targeted theranostic gold nanoparticles for photosensitizer delivery in brain tumors. Nanoscale.

[365] Wang X., Li J., Wang Y., Koenig L., Gjyrezi A., Giannakakou P., Shin E.H., Tighiouart M., Chen Z.G., Nie S. (2011). A folate receptor-targeting nanoparticle minimizes drug resistance in a human cancer model. ACS Nano.

[366] Singh I., Swami R., Pooja D., Jeengar M.K., Khan W., Sistla R. (2016). Lactoferrin bioconjugated solid lipid nanoparticles: A new drug delivery system for potential brain targeting. J. Drug Target..

[367] Ao M., Xiao X., Ao Y. (2018). Low density lipoprotein modified silica nanoparticles loaded with docetaxel and thalidomide for effective chemotherapy of liver cancer. Braz. J. Med. Biol. Res..

[368] Shevtsov M.A., Nikolaev B.P., Yakovleva L.Y., Dobrodumov A.V., Zhakhov A.V., Mikhrina A.L., Pitkin E., Parr M.A., Rolich V.I., Simbircev A.S. (2015). Recombinant interleukin-1 receptor antagonist conjugated to superparamagnetic iron oxide nanoparticles for theranostic targeting of experimental glioblastoma. Neoplasia.

[369] Abdellatif A.A.H., Aldalaen S.M., Faisal W., Tawfeek H.M. (2018). Somatostatin receptors as a new active targeting sites for nanoparticles. Saudi Pharm. J..

[370] Nikanjam M., Blakely E.A., Bjornstad K.A., Shu X., Budinger T.F., Forte T.M. (2007). Synthetic nano-low density lipoprotein as targeted drug delivery vehicle for glioblastoma multiforme. Int. J. Pharm..

[371] Thaxton C.S., Daniel W.L., Giljohann D.A., Thomas A.D., Mirkin C.A. (2009). Templated spherical high density lipoprotein nanoparticles. J. Am. Chem. Soc..

[372] Gutjahr A., Phelip C., Coolen A.-L., Monge C., Boisgard A.-S., Paul S., Verrier B. (2016). Biodegradable polymeric nanoparticles-based vaccine adjuvants for lymph nodes targeting. Vaccines.

[373] Marques Neto L.M., Kipnis A., Junqueira-Kipnis A.P. (2017). Role of metallic nanoparticles in vaccinology: Implications for infectious disease vaccine development. Front. Immunol..

[374] Shetab Boushehri M.A., Abdel-Motalleb M.M., Beduneau A., Pellequer Y., Lamprecht A. (2018). A nanoparticle-based approach to improve the outcome of cancer immunotherapy with lipopolysaccharides. Drug Deliv..

[375] Zolnik B.S., González-Fernández A., Sadrieh N., Dobrovolskaia M.A. (2010). Nanoparticles and the immune system. Endocrinology.

[376] Zhu M., Wang R., Nie G. (2014). Applications of nanomaterials as vaccine adjuvants. Hum. Vaccines Immunother..

[377] Dobrovolskaia M.A., McNeil S.E. (2007). Immunological properties of engineered nanomaterials. Nat. Nanotechnol..

[378] Gustafson H.H., Holt-Casper D., Grainger D.W., Ghandehari H. (2015). Nanoparticle uptake: The phagocyte problem. Nano Today.

[379] Yang E.-J., Choi I.-H. (2013). Immunostimulatory effects of silica nanoparticles in human monocytes. Immune Netw..

[380] Baron L., Gombault A., Fanny M., Villeret B., Savigny F., Guillou N., Panek C., Le Bert M., Lagente V., Rassendren F. (2015). The NLRP3 inflammasome is activated by nanoparticles through ATP, ADP and adenosine. Cell Death Dis..

[381] Hu Q., Zhao F., Guo F., Wang C., Fu Z. (2017). Polymeric nanoparticles induce NLRP3 inflammasome activation and promote breast cancer metastasis. Macromol. Biosci..

[382] Petersen L.K., Ramer-Tait A.E., Broderick S.R., Kong C.-S., Ulery B.D., Rajan K., Wannemuehler M.J., Narasimhan B. (2011). Activation of innate immune responses in a pathogen-mimicking manner by amphiphilic polyanhydride nanoparticle adjuvants. Biomaterials.

[383] Camacho A.I., Da Costa Martins R., Tamayo I., de Souza J., Lasarte J.J., Mansilla C., Esparza I., Irache J.M., Gamazo C. (2011). Poly(methyl vinyl ether-co-maleic anhydride) nanoparticles as innate immune system activators. Vaccine.

[384] Uto T., Akagi T., Yoshinaga K. (2011). The induction of innate and adaptive immunity by biodegradable poly(g-glutamic acid) nanoparticles via a TLR4 and MyD88 signaling pathway. Biomaterials.

[385] Kedmi R., Ben-Arie N., Peer D. (2010). The systemic toxicity of positively charged lipid nanoparticles and the role of Toll-like receptor 4 in immune activation. Biomaterials.

[386] Shetab Boushehri M.A., Stein V., Lamprecht A. (2018). Cargo-free particles of ammonio methacrylate copolymers: From pharmaceutical inactive ingredients to effective anticancer immunotherapeutics. Biomaterials.

[387] De Groot A.M., Thanki K., Gangolff M., Falkenberg E., Zeng X., van Bijnen D.C., van Eden W., Franzyk H., Nielsen H.M., Broere F. (2018). Immunogenicity testing of lipidoids in vitro and in silico: Modulating lipidoid-Mediated TLR4 activation by nanoparticle design. Mol. Ther. Nucleic Acids.

[388] Tanaka T., Legat A., Adam E., Steuve J., Gatot J.-S., Vandenbranden M., Ulianov L., Lonez C., Ruysschaert J.-M., Muraille E. (2008). DiC14-amidine cationic liposomes stimulate myeloid dendritic cells through Toll-like receptor 4. Eur. J. Immunol..

[389] Lonez C., Irvine K.L., Pizzuto M., Schmidt B.I., Gay N.J., Ruysschaert J.-M., Gangloff M., Bryant C.E. (2015). Critical residues involved in Toll-like receptor 4 activation by cationic lipid nanocarriers are not located at the lipopolysaccharide-binding interface. Cell. Mol. Life Sci..

[390] Qu G., Liu S., Zhang S., Wang L., Wang X., Sun B., Yin N., Gao X., Xia T., Chen J.-J. (2013). Graphene oxide induces Toll-like Receptor 4 (TLR4)-dependent necrosis in macrophages. ACS Nano.

[391] Bianchi M.G., Allegri M., Costa A.L., Blosi M., Gardini D., Del Pivo C., Prina-Mello A., Di Cristo L., Bussolati O., Bergamaschi E. (2015). Titanium dioxide nanoparticles enhance macrophage activation by LPS through a TLR4-dependent intracellular pathway. Toxicol. Res..

[392] Huang C., Sun M., Yang Y., Wang F., Ma X., Li J., Wang Y., Ding Q., Ying H., Song H. (2017). Titanium dioxide nanoparticles prime a specific activation state of macrophages. Nanotoxicology.

[393] Demento S.L., Eisenbarth S.C., Foellmer H.G., Platt C., Caplan M.J., Mark Saltzman W., Mellman I., Ledizet M., Fikrig E., Flavell R.A. (2009). Inflammasome-activating nanoparticles as modular systems for optimizing vaccine efficacy. Vaccine.

[394] Sunshine J.C., Green J.J. (2013). Nanoengineering approaches to the design of artificial antigen-presenting cells. Nanomedicine.

[395] Hickey J.W., Vicente F.P., Howard G.P., Mao H.-Q., Schneck J.P. (2017). Biologically inspired design of nanoparticle artificial antigen-presenting cells for immunomodulation. Nano Lett..

[396] Neal L.R., Bailey S.R., Wyatt M.M., Bowers J.S., Majchrzak K., Nelson M.H., Haupt C., Paulos C.M., Varela J.C. (2017). The basics of artificial antigen presenting cells in T cell-based cancer immunotherapies. J. Immunol. Res. Ther..

[397] Eggermont L.J., Paulis L.E., Tel J., Figdor C.G. (2014). Towards efficient cancer immunotherapy: Advances in developing artificial antigen-presenting cells. Trends Biotechnol..

[398] Meyer R.A., Sunshine J.C., Perica K., Kosmides A.K., Aje K., Schneck J.P., Green J.J. (2015). Biodegradable nanoellipsoidal artificial antigen presenting cells for antigen specific T-cell activation. Small Weinh. Bergstr. Ger..

[399] Perica K., De León Medero A., Durai M., Chiu Y.L., Bieler J.G., Sibener L., Niemöller M., Assenmacher M., Richter A., Edidin M. (2014). Nanoscale artificial antigen presenting cells for T cell immunotherapy. Nanomed. Nanotechnol. Biol. Med..

[400] Agrahari V., Agrahari V. (2018). Facilitating the translation of nanomedicines to a clinical product: Challenges and opportunities. Drug Discov. Today.

[401] Sarmento B. (2019). Have nanomedicines progressed as much as we’d hoped for in drug discovery and development?. Expert Opin. Drug Discov..

[402] Agrahari V., Hiremath P. (2017). Challenges associated and approaches for successful translation of nanomedicines into commercial products. Nanomedicine.

[403] Shetab Boushehri M.A., Lamprecht A. (2015). Nanoparticles as drug carriers: Current issues with In Vitro testing. Nanomedicine.

[404] Lorscheidt S., Lamprecht A. (2016). Safety assessment of nanoparticles for drug delivery by means of classic in vitro assays and beyond. Expert Opin. Drug Deliv..

[405] Hussain S.M., Braydich-Stolle L.K., Schrand A.M., Murdock R.C., Yu K.O., Mattie D.M., Schlager J.J., Terrones M. (2009). Toxicity evaluation for safe use of nanomaterials: Recent achievements and technical challenges. Adv. Mater..

